# Evaluation of mycoparasitic *Trichoderma atroviride* and entomopathogenic *Aspergillus niger* as potential bioinsecticides against the dengue vector, *Aedes aegypti*

**DOI:** 10.3389/fcimb.2025.1502579

**Published:** 2025-04-10

**Authors:** A. V. Ranthilini C. Banduwardena, B. Anushka N. Mendis, Kasun M. Thambugala, H. Sachini D. Fernando, Itthayakorn Promputtha

**Affiliations:** ^1^ Department of Biology, Faculty of Science, Chiang Mai University, Chiang Mai, Thailand; ^2^ Center for Biotechnology, Department of Zoology, Faculty of Applied Sciences, University of Sri Jayewardenepura, Nugegoda, Sri Lanka; ^3^ Genetics and Molecular Biology Unit, Faculty of Applied Sciences, University of Sri Jayewardenepura, Nugegoda, Sri Lanka; ^4^ Center for Plant Materials and Herbal Product Research, University of Sri Jayewardenepura, Nugegoda, Sri Lanka; ^5^ Environmental Science Research Center (ESRC), Chiang Mai University, Chiang Mai, Thailand; ^6^ Natural Extracts and Innovative Products for Alternative Healthcare Research Group, Chiang Mai University, Chiang Mai, Thailand

**Keywords:** bioinsecticides, entomopathogenic fungi, mosquito control, dengue, *Aedes aegypti*

## Abstract

**Introduction:**

Over the past three decades, dengue disease incidence has significantly increased worldwide, creating serious public health concerns. The principal mosquito vector, *Aedes aegypti*, exhibits resistance to commonly used insecticides, reducing the efficacy of vector control measures. Thus, the necessity for alternate strategies is critical. Using bioinsecticides such as entomopathogenic fungi (EPF) is one such strategy. This study details the evaluation of mycoparasitic *Trichoderma atroviride* and entomopathogenic *Aspergillus niger* against pyrethroid-resistant and pyrethroid-susceptible *Ae. aegypti* populations.

**Materials and methods:**

Molecular identification of the isolated entomopathogenic fungal strains was done using ITS-rDNA sequence data. Larvicidal and adulticidal assays were performed using different spore concentrations of fungal species. Pupal emergence was assessed from the survived larvae of larvicidal assays.

**Results:**

Larvicidal assays revealed the highest mortality of 60% for *T*. *atroviride* after 9 days of exposure when compared with the highest mortality of 52% for *A*. *niger* after 6 days of exposure. No significant difference was observed between the pyrethroid-resistant and pyrethroid-susceptible mosquito colonies, suggesting a lack of connection between prior resistance status and EPF pathogenicity. No pupal mortality was observed, although pupal duration was prolonged. Both EPF strains exhibited 100% mortality in adulticidal assays, signifying the potential use of the two fungal species as adulticides.

**Conclusion:**

However, further studies are needed to understand the biology of EPF, its mechanism of action, the mosquito immune pathways activated, and the effect on non-target organisms. The findings have implications for the possible use of *A*. *niger* and *T*. *atroviride* as potential bioinsecticides against the control of *Ae. aegypti.*

## 1 Introduction

Dengue is a hyperendemic arboviral disease affecting tropical and subtropical regions of the world, with an estimated 100–400 million new infections occurring annually worldwide (Bhatt et al., 2013; WHO, 2024). The exponential rise of global dengue incidence rates has been attributed to rising environmental temperatures, urbanization, and human mobility (Paz-Bailey et al., 2024). The clinical manifestations of dengue range from asymptomatic or mild fever-like symptoms to severe life-threatening disease known as dengue hemorrhagic fever/dengue shock syndrome requiring hospitalizations (Tayal et al., 2023). High prevalence and frequent epidemics of dengue place a huge burden on a country’s healthcare system and economy due to hospitalizations, death, and lost productivity (Chen et al., 2024). For instance, Sri Lanka has been afflicted by dengue and dengue hemorrhagic fever epidemics for more than 40 years since the mid-1960s (Sirisena and Noordeen, 2014). The average hospitalization costs for dengue during the epidemic year of 2012 at secondary care hospitals ranged from US$216 to US$609 per pediatric case and US$196 to US$866 per adult case in Colombo district, Sri Lanka (Thalagala et al., 2016). The total number of dengue cases reported in the year 2023 is 88,458, with the district of Colombo having a notable number of occurrences, accounting for almost 21% of all cases. The reported cases increased to 76,467 in 2022 from 36,120 in 2021 (Mendis et al., 2024).

Mosquitoes belonging to the genus *Aedes* are the vectors of the dengue virus, with *Aedes aegypti* being considered as the main vector. *Aedes aegypti* is a major public health concern due to its ability to transmit approximately 30 of the hundreds of known arthropod-borne viruses, or arboviruses, that cause diseases in people including four vital arboviruses, namely, dengue (DENVs), chikungunya (CHIKV), Zika (ZIKV), and yellow fever (YFV) viruses. *Aedes aegypti* is an anthropophilic and endophilic mosquito that lives in close proximity to human habitations and breeds frequently in man-made containers (Facchinelli et al., 2023).

In the absence of a successful preventive vaccine or antiviral therapy, the control of the disease spread is achieved through vector population control via eradication of breeding sites and using chemical insecticides (Wilson et al., 2020). However, the large number and the vast variety of breeding sites together with the development of resistance in the *Ae. aegypti* mosquito populations to commonly used chemical insecticides coupled with toxicity to non-target organisms have rendered the currently employed control methods inefficient (Accoti et al., 2021). Thus, the necessity of implementing alternate methods for controlling mosquitoes is being emphasized. Several vector control tools and strategies are being developed, including new insecticides using botanicals, genetically engineered organisms, and entomopathogenic organisms (Achee et al., 2019). Although the use of botanicals as insecticides has been successfully experimented with, the rapid biodegradation of botanical active ingredients in field conditions and the shortage of plant biomass for the continuous production of insecticides have become major obstacles in commercialization (Isman, 2020). Although genetically engineered organisms have been successfully experimented and implemented (Wang et al., 2021), they are expensive to produce and have resulted in complex socio-political and ethical issues (Meghani and Boëte, 2018). The use of entomopathogenic organisms is an environmentally friendly alternative vector control strategy that enables the possible use of fungi, viruses, and bacteria as biological insecticides (Qin et al., 2023).

The term “entomopathogenic fungi (EPF)” refers to parasitic microorganisms that have the ability to infect arthropods (Islam et al., 2021). Entomopathogenic fungi infect a wide range of life stages in insects, ranging from eggs to adults. Entomopathogenic fungi can either invade their host directly through the cuticle and grow inside their body, or EPF can produce a wide variety of extracellular enzymes, thus degrading the integument of the host organism (Brivio and Mastore, 2020; Shen et al., 2019; Souza-Neto et al., 2019). The use of EPF for mosquito control is very appealing as a control agent due to several reasons. They can infect mosquitoes directly through contact with their cuticles, without the need for ingestion, and they can be applied to control any stage of the life cycle of mosquitoes. However, repeated application, being most recognized to control larvae, is not mandatory due to the ability of the fungus to produce new spores on the dead mosquito body and disseminate (Qin et al., 2023).

Several species of EPF are commonly used and commercialized for the biological control of forest and agricultural pests (Lacey et al., 2015; Sharma et al., 2023). The entomopathogenic fungi *Beauveria bassiana* and *Metarhizium anisopliae* are the most characterized and widely used fungi, and many species and subspecies of these two fungi have been commercially formulated to be used as insecticides for a variety of agricultural insects (Mascarin et al., 2019; Yapa et al., 2024). Due to their environmentally benign methods and potential for eliminating all mosquito life stages, numerous studies have also indicated their promise for managing disease-carrying mosquito vectors (Rodrigues-Alves et al., 2020).

In Sri Lanka, EPF are heavily understudied as mosquito control agents. This study aims to discover the larvicidal and the adulticidal potential of the mycoparasitic *Trichoderma atroviride* and the entomopathogenic *Aspergillus niger* for the control of *Ae. aegypti* mosquito vector. For the first time in Sri Lanka, the effect of different conidia concentrations and time of exposure on highly pyrethroid-resistant and pyrethroid-susceptible mosquito populations was tested for two locally isolated fungi species.

## 2 Materials and methods

### 2.1 Fungal strains

Two fungal isolates were used for the current study. One strain was isolated from an *Ae. aegypti* mosquito cadaver by culturing the sample onto a potato dextrose agar (PDA) (pH = 5.65) medium followed by a subculturing step (GMBUCC 24-015). Morphological identification of the isolated fungal strain was carried out according to Diba et al. (2007). The mycoparasitic strain of *T. atroviride* (GMBUCC 24-014) was obtained from the culture collection of the Genetics and Molecular Biology Unit, University of Sri Jayewardenepura, Sri Lanka (Konara et al., 2024), as the second isolate. Both fungal isolates were cultured at room temperature conditions [27°C ± 2°C; relative humidity (RH) ≥ 80%].

### 2.2 Molecular identification of entomopathogenic fungi

The DNA extraction of each fungal isolate was performed following the method of Thambugala et al. (2015). The nuclear ribosomal internal transcribed spacers region (ITS) was amplified using the primers ITS4 and ITS5 (White et al., 1990).

The amplification reactions were carried out in a thermal cycler, with each 25 μl of reaction mixture consisting of 12.5 μl of FastGene Master Mix (1.5 mM of MgCl_2_ at 1×) (Nippon Genetics, Europe), 0.25 μl each of the forward and reverse primers, and 5 ng of the DNA template. The polymerase chain reaction (PCR) cycling conditions included an initial denaturation step at 95°C for 3 min, followed by 30 cycles of denaturation at 94°C for 1 min, annealing at 54°C for 30 s, and extension at 72°C for 1 min. A final elongation step was performed at 72°C for 10 min, followed by a holding step at 4°C (Thambugala et al., 2017).

Successfully amplified products were sent to Macrogen Inc. (Korea) for Sanger sequencing, and the returned sequences were blasted in the NCBI database (https://blast.ncbi.nlm.nih.gov/Blast.cgi?PROGRAM=blastn&PAGE_TYPE=BlastSearch&LINK_LOC=blasthome) against fungal taxa for species identification. The new sequences generated in this study were deposited in GenBank.

### 2.3 Entomopathogenic fungi suspensions

Conidia were harvested from each fungal species separately by scraping the sporulated colonies on the surface of the agar and suspended into respective conical flasks containing sterile filter water with 0.05% Tween 20 (v/v aqueous solution). The resulting conidial suspensions were filtered separately through sterile filter paper (Whatman No. 1) to obtain a hyphal-free conidial suspension. The suspension containing conidia was concentrated by removing the supernatant after centrifugation for 3 min at 5,000 rpm. The conidial concentration of the suspension was determined using a hemocytometer and the desired concentrations (1 × 10^6^, 1 × 10^7^, 1 × 10^8^, 1 × 10^9^, and 1 × 10^10^ conidia/ml) were prepared by making adjustments using sterile filtered water with 0.05% Tween 20 (v/v aqueous solution) (Renuka et al., 2023; Scholte et al., 2003).

### 2.4 Mosquito rearing

Two mosquito populations, a pyrethroid-susceptible mosquito population (New Orleans mosquito colony, NeO) and a pyrethroid-resistant mosquito population (USJ mosquito colony), were used for the present study. Both mosquito colonies were reared within the insectary at the Department of Zoology, University of Sri Jayewardenepura, Sri Lanka. Dr. William C. Black IV, Colorado State University, USA, initially donated eggs of the NeO mosquito strain. This strain is 100% susceptible to pyrethroid insecticides and, thus, was used as the insecticide-susceptible strain. A pyrethroid-resistant colony was established from mosquito eggs collected from the University of Sri Jayewardenepura, Sri Lanka premises, where a highly pyrethroid-resistant mosquito population has been recorded previously (Fernando et al., 2018, 2020; Mendis et al., 2024). Mosquito eggs for the USJ population were collected using ovitraps placed in the indoor environment of the sampling location—the University of Sri Jayewardenepura. The traps were collected after 5 days, along with the larval stages already present in the ovitraps’ water at the time of collection. Eggs were hatched in deoxygenated water. All immature stages were fed on sterile fish feed (Fernando et al., 2020). Upon emergence, adult mosquitoes were morphologically classified to the species level using standard taxonomic keys (Rueda, 2004). To induce egg-laying, female *Ae. aegypti* were artificially blood-fed and mated with 10% sucrose-fed male *Ae. aegypti*. The insectary held the mosquito cages at 27°C ± 2°C room temperature, 80%–100% RH, and a 12-h:12-h light:dark photoperiod. The process was continued for several generations to obtain adequate population numbers required for the bioassays. The same rearing procedure was followed for the NeO *Ae. aegypti* mosquito population (Fernando et al., 2020).

Both mosquito populations (NeO and USJ) were continuously tested for pyrethroid susceptibility using the WHO standard susceptibility tube bioassays (WHO, 2022). The insecticide susceptibility status for each colony was determined according to the WHO criteria, whereby a final mortality of 98%–100% indicates full susceptibility, while mortality lower than 97% suggests resistance in the colony.

### 2.5 Larvicidal bioassays

Larvicidal bioassays of the EPF were performed following the methods described by Vivekanandhan et al. (2020). Twenty-five second instar larvae of *Ae. aegypti* from each colony (25 sets from each USJ and NeO population) were put into disposable containers (11 cm height, 6 cm diameter), containing 250 ml of tap water and 250 ml from each of the five different fungal spore concentrations (1 × 10^6^, 1 × 10^7^, 1 × 10^8^, 1 × 10^9^, and 1 × 10^10^ conidia/ml). An equal number of controls were set up simultaneously using sterile distilled water + 0.05% Tween 20. The mortality of the exposed mosquito larvae was assessed for 12 days, starting from the second day of treatment (Vivekanandhan et al., 2020). Larvae that failed to move after probing with a needle were identified as dead larvae. The experiment was replicated four times for each spore concentration per fungal species at 27°C ± 2°C room temperature and 80%–100% RH.

### 2.6 Adulticidal bioassays

For each population (NeO and USJ), adulticidal bioassays were carried out using the WHO standard bioassay kits. Adult *Ae. aegypti* mosquitoes that were 2 to 5 days old, which were sugar-fed, were used in the experiment.

Whatman No. 1 filter papers were cut to the dimensions of the WHO standard bioassay kit (8 × 6 cm) and sterilized by autoclaving at 121°C for 15 min. The sterilized filter paper pieces were placed on large petri dishes. One milliliter from each conidia solution with concentrations of 1 × 10^6^, 1 × 10^7^, 1 × 10^8^, 1 × 10^9^, and 1 × 10^10^ conidia/ml was pipetted evenly on five separate sterilized filter paper pieces using a sterilized pipette. The filter paper piece designated for the negative control was prepared by pipetting out a solution made up of sterile distilled water + 0.05% Tween 20. All were left to dry for 48 h at 80% RH condition. The dried filter paper pieces were gently placed in the WHO standard bioassay kits under sterile conditions so that the paper coated the inside of the tubes (Scholte et al., 2003). Accordingly, six exposure tubes were prepared (for five spore concentrations and the negative control).

Each holding tube containing 25 healthy adult mosquitoes was examined carefully for any damage before being transferred to exposure tubes. The gender ratio of mosquitoes was 4:1 (female:male). The bioassays were carried out at 27°C ± 2°C (Scholte et al., 2007). Following the exposure for 1 h, the adult mosquitoes were reintroduced to the holding tubes, where they were fed with a 10% sucrose solution (Accoti et al., 2021). The knocked-down mosquitoes were recorded 1 h after exposure, followed by 24-h intervals, for a period of 12 days.

The experiment was replicated four times for each spore concentration per fungal species at 27°C ± 2°C room temperature and 80%–100% RH.

### 2.7 Confirmation of the pathogenicity of the fungi

Dead mosquitoes resulting from the bioassay experiments were randomly selected, and fungi were re-isolated and cultured on PDA medium at room temperature conditions (27°C ± 2°C; RH ≥ 80%) for the confirmation of entomopathogenic activity.

### 2.8 Post-experimental treatment

World Health Organization standard bioassay kits and disposable containers used throughout the experimental procedure were sterilized by dipping in a 95% ethanol bath. All glassware was autoclaved at 121°C for 15 min. The used filter paper pieces contaminated with fungal conidia were autoclaved at 121°C for 15 min and disposed of as litter.

### 2.9 Statistical analysis

The comparison of the mean mortality of each adult and larval population and the pupal emergence of survived larvae from larvicidal assays exposed to the spore concentrations from the two fungal isolates was analyzed using analysis of variance (ANOVA). Tukey’s pairwise comparison with Bonferroni correction was done for each significant group. The significance of each of the spore concentrations applied and time intervals of exposure, toward the cumulative larval and adult mortalities and pupal emergence for each mosquito population treated with the two fungal species, was determined using Kruskal–Wallis pairwise multiple comparison. The *p*-value was adjusted using the false discovery rate (FDR) followed by a Dunn pairwise analysis with the “ggbetweenstats” function. The plots were prepared using version 4.3.3 of the R statistical software (R Development Core Team, 2024). Lethal parameters, including LC_50_ (the lethal concentration required to kill 50% of the population), LC_90_ (the lethal concentration required to kill 90% of the population), and LT_50_ (the lethal time required to kill 50% of the population at a specified concentration), were determined using probit analysis.

## 3 Results

### 3.1 Identification of fungal strains

The fungal isolate from the mosquito cadaver was identified morphologically and confirmed using the 600-bp genomic DNA fragment amplified using the ITS region. The strain GMBUCC 24-015 (GenBank accession number: PQ226004) was identified as *A. niger* based on the closest matches in the BLAST search (strain number QMS6; GenBank accession number: MT430878; percent identity 99.16%, query coverage 99%).

### 3.2 Cumulative mortality percentages of *Aedes aegypti* larvae to different spore concentrations of fungi over time

**Figure 1:**
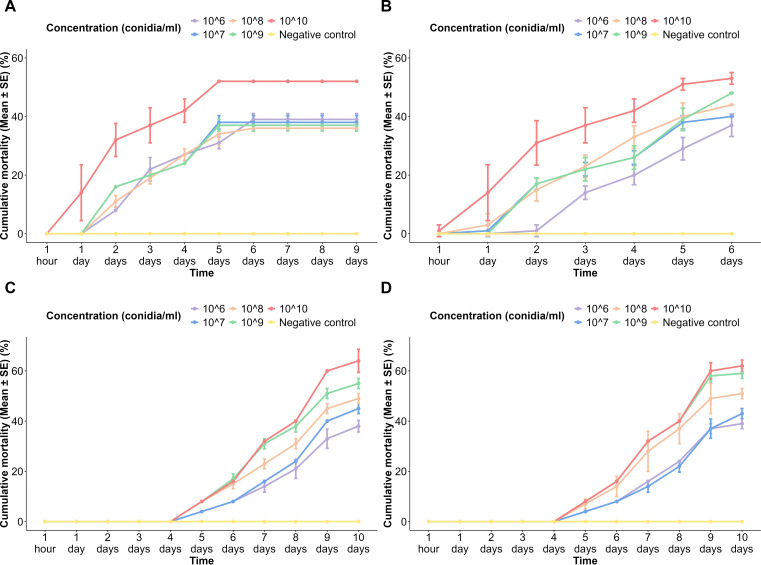
Cumulative mortality percentages of *Aedes aegypti* larvae to different spore concentrations of fungi over time. **(A)** *Aspergillus niger* on the USJ population, **(B)** *A. niger* on the NeO population, **(C)** *Trichoderma atroviride* on the USJ population, and **(D)** *T. atroviride* on the NeO population.

In the USJ mosquito population exposed to spores of *A. niger*, the highest cumulative mortality percentage of 52% ± 0% was observed at the highest spore concentration (1 × 10^10^ conidia/ml). In contrast, all the other concentrations showed a maximum cumulative mortality percentage ranging from 37% ± 1% to 39% ± 1% (**Figure 1A**). All spore concentrations were devoid of any larval death beyond the 6th day. In the NeO population exposed to *A*. *niger* spores, the highest cumulative mortality recorded was 53% ± 0%, which was recorded at the highest spore concentration (1 × 10^10^ conidia/ml) (**Figure 1B**). A positive correlation was observed between the concentration of spores applied and the cumulative mortality percentage of the mosquito population over time, as indicated by the ANOVA results (*p* < 2e−16).

The effect of *T*. *atroviride* spores on the USJ mosquito population showed a delayed mortality, with onset occurring after 4 days of exposure. The highest cumulative mortality reached 60% ± 1.63% at the highest spore concentration (1 × 10^10^ conidia/ml) (**Figure 1C**). Similarly, upon treatment of the *T*. *atroviride* spores on the NeO larval population, mortality onset occurred after 4 days of exposure. The highest cumulative mortality, 60% ± 3.27%, was recorded at the spore concentration of 1 × 10^10^ conidia/ml (**Figure 1D**). No larval death was observed after 10 days of exposure in any concentration of *T*. *atroviride* spores in both *Ae. aegypti* larval populations (**Figure 1**).

Tukey’s pairwise comparisons revealed a significant difference between the time intervals (*p* < 2 × 10^−16^), fungal spore concentrations (*p* < 2 × 10^−16^), and fungal species (*p* < 2 × 10^−16^). When comparing different time intervals, significant differences were observed except for a few, such as between 1 h and 24 h, 1 h and 48 h, 24 h and 48 h, 48 h and 72 h, 48 h and 96 h, and 72 h and 96 h. A significant difference was observed between different spore concentrations other than 1 × 10^9^ and 1 × 10^10^, 1 × 10^7^ and 1 × 10^6^, 1 × 10^8^ and 1 × 10^6^, 1 × 10^9^ and 1 × 10^6^, 1 × 10^8^ and 1 × 10^7^, 1 × 10^9^ and 1 × 10^7^, and 1 × 10^8^ and 1 × 10^9^ conidia/ml. A significant difference between the larval mortalities of the two species *A*. *niger* and *T*. *atroviride* was also observed.

### 3.3 Cumulative pupal emergence of *Aedes aegypti* to different spore concentrations of fungi over time

**Figure 2:**
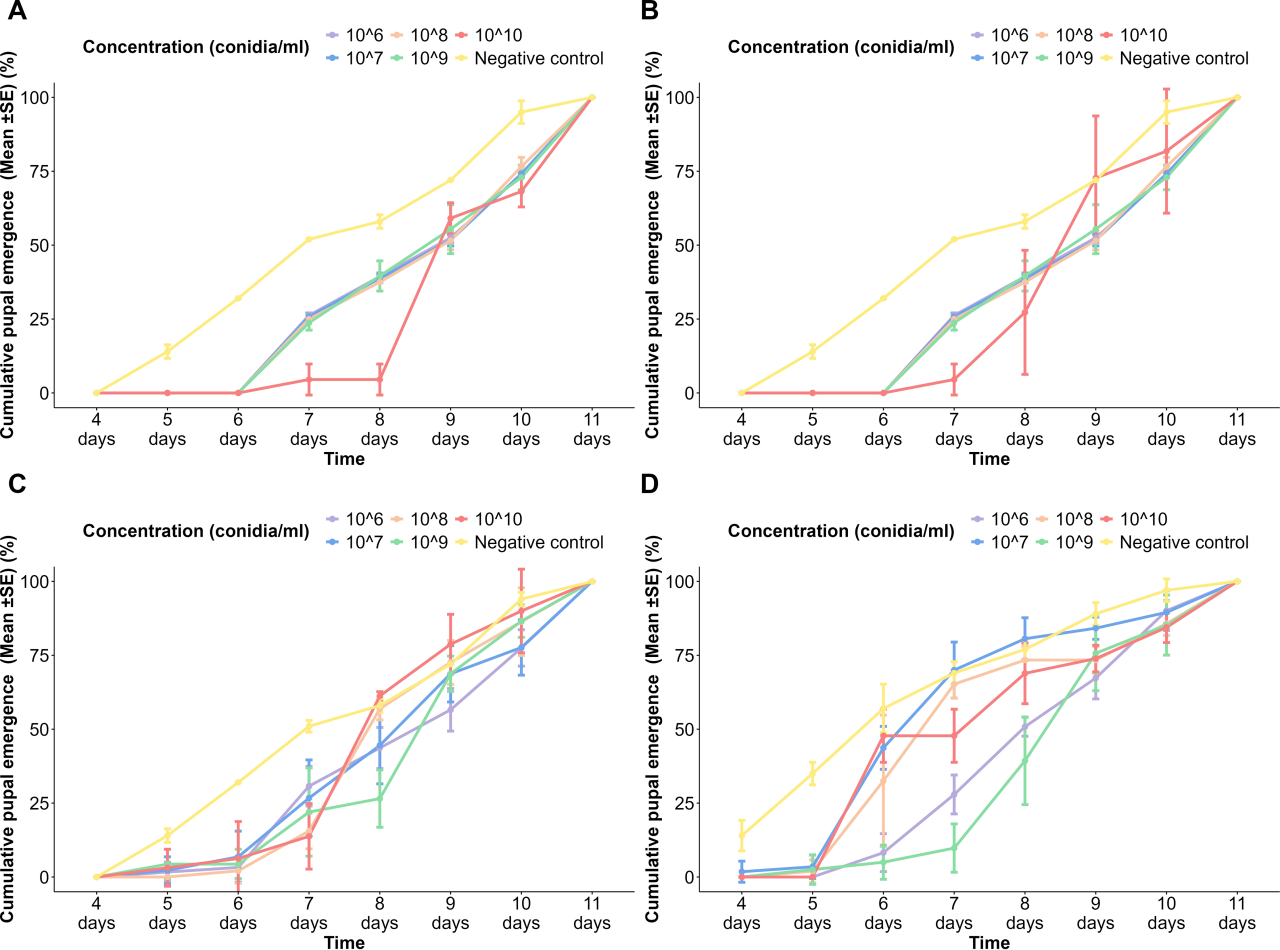
Cumulative pupal emergence *of Aedes aegypti* to different spore concentrations of fungi over time. **(A)** *Aspergillus niger* on the USJ population, **(B)** *A*. *niger* on NeO population, **(C)** *Trichoderma atroviride* on the USJ population, and **(D)** *T. atroviride* on the NeO population.

More than 50% pupal emergence occurred by the 9th day in the *A*. *niger* spore-treated population, while the negative control reached 50% pupal emergence by the 7th day (**Figure 2A**). All the concentrations (1 × 10^6^, 1 × 10^7^, 1 × 10^8^, 1 × 10^9^ conidia/ml) except 1 × 10^10^ conidia/ml of the *A*. *niger* spore-treated NeO population showed a similar pattern of pupal emergence to that of the USJ population (**Figure 2B**). Nevertheless, the highest concentration of the spores, 1 × 10^10^ conidia/ml, exhibited a significantly higher rate of pupal emergence in NeO after the 7th day compared to the USJ population (**Figure 2A**). Specifically, on the 8th day, 27.3% ± 21% of the surviving larvae had pupated in the NeO population, whereas only 4.55% ± 5.25% had pupated in the USJ population.

In both USJ (**Figure 2C**) and NeO (**Figure 2D**) populations treated with *T*. *atroviride* spores, the pupal emergence was slightly delayed but still reached 100% at day 11 at the highest concentration (1 × 10^10^ conidia/ml). Lower concentrations (1 × 10^6^ to 1 × 10^9^ conidia/ml) followed a similar trend with cumulative pupal emergence increasing over time but generally lower than the control initially. Until the 6th day, the control group of the NeO population exhibited a significant increase in the pupation of the treated groups. From the 7th day, the pupation of different concentrations became noticeable. The negative control continued to increase steadily, while the increasing rate varied among the treated groups. By the 11th day, 100% pupation was observed in all the treated groups.

The plots indicated that fungal spore concentrations affect the cumulative pupal emergence of *Aedes* larvae, with higher concentrations showing a slight delay in pupal emergence compared to the control. However, at the end of the observation period, cumulative pupal emergence across all treatment approaches 100% pupation. In all negative control groups, pupal emergence commenced on the 5th day, while by the 11th day, pupation had completed. The plot indicated a slight delay in the treated populations when compared with the control groups. In all treated populations, the pupation commenced on day 6, showing a delay compared to the control groups, while completed on day 11 (**Figures 2C, D**). Significantly, no larval death (100% pupation) occurred in the tested populations, as well as in the controls (**Figure 2**).

There was a significant difference in the pupal emergence when the following variables were considered: time (*p* < 2 × 10^−16^), spore concentration (*p* < 2 × 10^−16^), fungal species (*p* < 2 × 10^−16^), and population of *Ae. aegypti* (*p* < 3.64 × 10^−10^). Tukey’s pairwise comparisons revealed a significant difference in pupation between emergence between the negative control and all the other concentrations. Tukey’s pairwise comparison revealed that there is a significant difference in pupal emergence with the fungal species (*p* = 0.0005456) and the *Ae. aegypti* population (*p* = 0.0448608).

### 3.4 Cumulative mortality of *Aedes aegypti* adults to different spore concentrations of fungi over time

**Figure 3:**
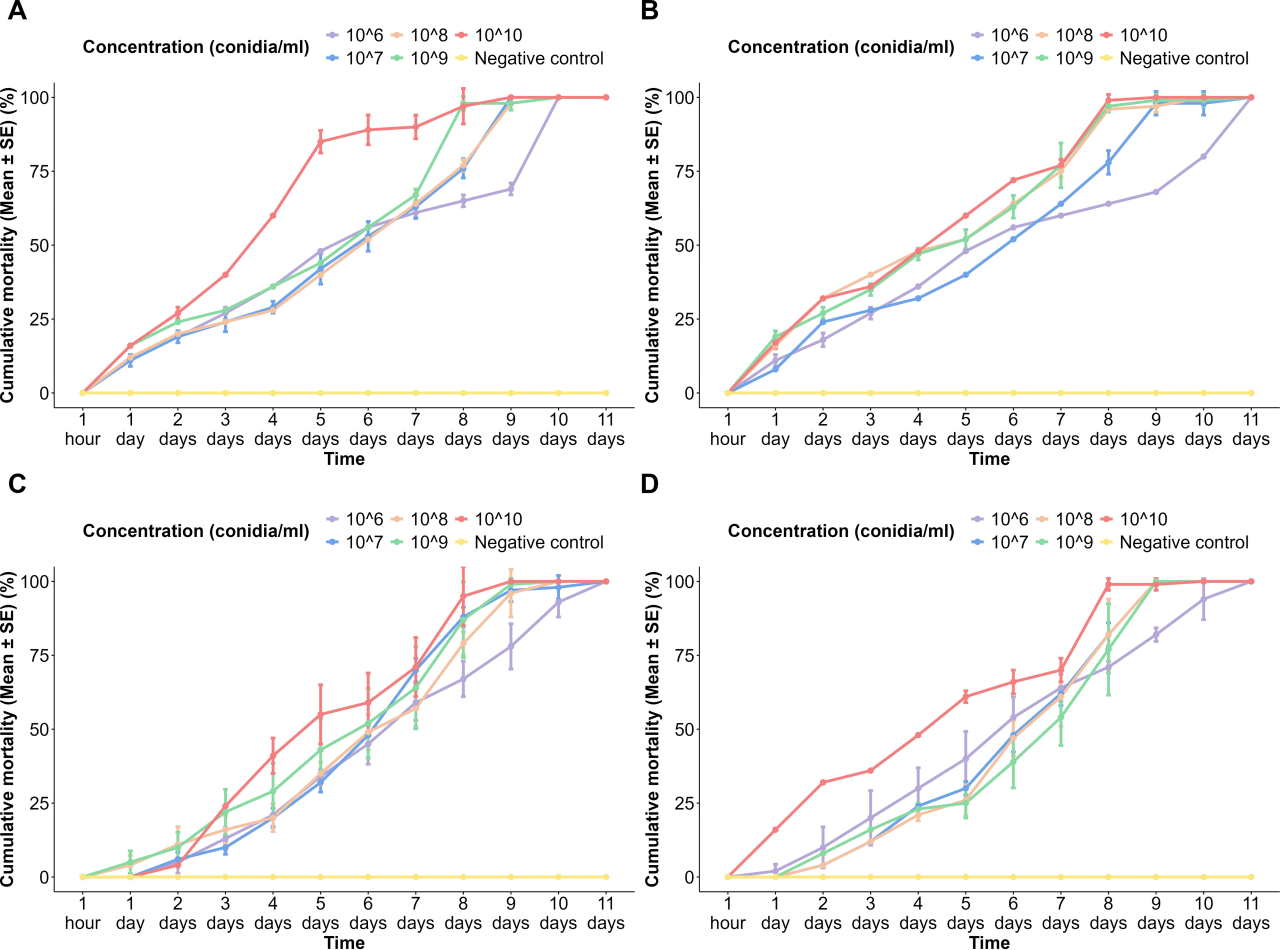
Cumulative mortality of *Aedes aegypti* adults to different spore concentrations of fungi over time. **(A)** *Aspergillus niger* on the USJ population, **(B)** *A. niger* on the NeO population, **(C)** *Trichoderma atroviride* on the USJ population, and **(D)** *T. atroviride* on the NeO population.

In the USJ adult population treated with spores of *A*. *niger*, at 24 h of treatment, mortality had onset in all populations except the negative control. Within the treated groups, there was a gradual increase in the cumulative mortality over time, and it reached 100% ± 0% in all concentrations. The highest spore concentration always exhibited a higher cumulative mortality rate than the other spore concentrations at each time interval (**Figure 3A**). At 24 h of treatment with *A*. *niger* spores, all concentrations resulted in adult mortality in the NeO population. All concentrations showed nearly similar trends in mortalities except the lowest concentration (1 × 10^6^ conidia/ml), as it has taken a much longer time duration with slow rates of mortality (**Figure 3B**).

Upon application of *T*. *atroviride* spores on the USJ adult population, although 1 × 10^10^ conidia/ml concentration is the highest among others, its mortality occurred at 48 h of exposure, then continued to a sudden increase in mortality percentages until the 5th day of exposure (**Figure 3C**). All populations treated reached 100% mortality by the 11th day post-exposure (**Figures 3C, D**).

Mortality was first recorded in the population exposed to the lowest spore concentration (1 × 10^6^ conidia/ml) of *T*. *atroviride*. By 48 h, every treated population had recorded mortality. Nevertheless, the population treated with the 1 × 10^10^ conidia/ml concentration of *T*. *atroviride* showed a considerably higher cumulative mortality compared to the cumulative mortalities of other concentrations. There was no mortality recorded in any of the control groups (**Figure 3**).

There was a significant difference in the pupal emergence in terms of the following variables: time (*p* < 2 × 10^−16^), spore concentration (*p* < 2 × 10^−16^), and fungal species (*p* < 7.04 × 10^−10^). A significant difference in adult mortality was observed between the lower and higher conidia concentrations.

A significant difference was observed in Tukey’s pairwise comparisons among higher and lower time durations, including 1 h and 24 h and 24 h and 48 h. A positive correlation was observed between the spore concentration applied and the time taken to achieve the highest mortality.

Tukey’s pairwise comparisons revealed a significant difference in adult mortalities between the fungal species (*p* = 0.0135).

### 3.5 Kruskal–Wallis multiple comparisons

#### 3.5.1 Kruskal–Wallis multiple comparisons of larvicidal assay

The larval mortality among USJ population spores of *A. niger* showed a significant difference (*p* < 2.2 × 10^−16^) among different time intervals and spore concentrations. Most of the time intervals resulted in a significance in larval mortality when compared. The negative control and the highest spore concentration (1 × 10^10^ conidia/ml) exhibited a significant difference with the larval mortality recorded in all the other spore concentrations (**Supplementary Figure 1**).

There was a significant difference of the larval mortality among different time intervals (*p* < 2.2 × 10 ^−16^) and spore concentrations (*p* < 1.824 × 10^−10^) in the NeO population treated with *A*. *niger* spores. Most of the pairwise comparisons among the time intervals and spore concentrations were significant (**Supplementary Figure 2**).

The USJ population treated with *T. atroviride* spores showed a significant difference in larval mortality when compared among different time intervals (*p* < 2.2 × 10^−16^) and spore concentrations (*p* < 1.013 × 10^−7^). Only a few intervals including 1 h and 24 h and 24 h and 48 h were observed as non-significant, while in the pairwise comparison of concentrations, they appeared significantly different in larval mortalities except for several comparisons such as 1 × 10^6^ and 1 × 10^10^ and 1 × 10^7^ and 1 × 10^10^ conidia/ml (**Supplementary Figure 3**).

Larval mortalities resulting from all concentrations of *T. atroviride* spores (*p* < 2.2 × 10^−16^) and the time intervals (*p* < 1.024 × 10^−7^) applied to the NeO population appeared to be significant. Most of the pairwise comparisons of time intervals were significantly different in the larval mortality recorded, excluding a few such as 48 h and 96 h and 72 h and 96 h. All the spore concentrations had significantly different larval mortalities except for a few, such as 1 × 10^9^ and 1 × 10^10^, 1 × 10^8^ and 1 × 10^10^, 1 × 10^7^ and 1 × 10^10^, and 1 × 10^7^ and 1 × 10^10^ conidia/ml (**Supplementary Figure 4**).

#### 3.5.2 Kruskal–Wallis multiple comparisons of pupal emergence

The pupal emergence was significantly different between different time intervals (*p* < 2.2×10^−16^) (**Supplementary Figure 5**) in the study groups but was not significant between concentrations of spores (*p* > 0.05). It is quite evident that most of the pairwise comparisons of pupal emergence between the time intervals in the tested groups were significant (**Supplementary Figure 6**).

#### 3.5.3 Kruskal–Wallis multiple comparisons of adulticidal assays

All the *Ae. aegypti* populations (USJ and NeO) treated with spores of *A*. *niger* and *T*. *atroviride* showed a significant difference in the adult mortalities when compared between different time intervals (**Supplementary Figure 7**) and spore concentrations (**Supplementary Figure 8**) (*p* < 2.2 × 10^−16^).

### 3.6 LC_50_, LC_90_, and LT_50_ of larvicidal and adulticidal assays

The results of LC_50_, LC_90_, and LT_50_ values of the larvicidal and adulticidal assays are tabulated in **Tables 1**, **2**. The LC_50_ and LC_90_ values decreased with the increase of concentration most of the time, but there were some deviations. The adult populations showed high LC_50_ values at the beginning, which decreased over time due to the increase in susceptibility because of extended exposure. *Aspergillus niger* exhibits a decline in LC_50_ over time which attained 100% mortality at 264 h. The larval populations exhibited relatively slower mortality trends. At early time intervals (24–96 h), *T*. *atroviride* showed high LC_50_ values (**Table 1**).

**Table 1:** LC_50_ and LC_90_ values of larvicidal and adulticidal assays treated with *Aspergillus niger* and *Trichoderma atroviride* at each time interval.

Stage	Treated fungi	Time (h)	LC_50_	LC_90_
Adult	*Aspergillus niger*	24	2.17E+21	8.34E+36
		48	2.50E+16	4.67E+31
		72	2.63E+14	6.57E+30
		96	1.27E+10	4.05E+20
		120	6.26E+07	6.34E+16
		144	1.09E+06	3.88E+14
		168	4.76E+04	4.76E+12
		192	1.85E+05	1.34E+08
		216	1.29E+05	7.52E+06
		240	9.84E+03	7.94E+05
		264	0.00E+00	0.00E+00
Adult	*Trichoderma atroviride*	24	8.73E+14	5.83E+20
		48	4.39E+17	8.12E+26
		72	1.14E+15	6.47E+24
		96	6.93E+12	1.49E+23
		120	4.05E+10	3.23E+21
		144	5.02E+07	2.46E+29
		168	3.20E-04	1.44E+40
		192	3.23E+03	2.16E+09
		216	1.97E+04	3.35E+06
		240	1.94E+04	5.55E+05
		264	0.00E+00	0.00E+00
Larvae	*Aspergillus niger*	24	7.44E+12	9.88E+16
		48	1.33E+13	5.95E+19
		72	8.39E+13	2.11E+24
		96	1.43E+13	1.05E+25
		120	3.38E+10	7.67E+21
		144	2.09E+10	5.49E+25
		168	6.18E+11	5.10E+31
		192	6.18E+11	5.10E+31
		216	6.18E+11	5.10E+31
Larvae	*Trichoderma atroviride*	24	–	–
		48	–	–
		72	–	–
		96	–	–
		120	3.37E+23	2.55E+36
		144	3.70E+17	1.31E+28
		168	2.39E+12	1.09E+20
		192	2.20E+11	1.42E+20
		216	2.88E+08	1.16E+16
		240	8.28E+07	1.12E+16

**Table 2:** LT_50_ values of larvicidal and adulticidal assays treated with *Aspergillus niger* and *Trichoderma atroviride* at each concentration.

Stage	Treated fungi	Concentration (conidia/ml)	LT_50_ (h)
Adult	*Aspergillus niger*	1 × 10^6^	134.8945
		1 × 10^7^	124.86983
		1 × 10^8^	112.32565
		1 × 10^9^	106.45591
		1 × 10^10^	87.10856
Adult	*Trichoderma atroviride*	1 × 10^6^	144.8318
		1 × 10^7^	139.1343
		1 × 10^8^	139.9432
		1 × 10^9^	135.612
		1 × 10^10^	107.3398
Larvae	*Aspergillus niger*	1 × 10^6^	223.3184
		1 × 10^7^	231.8991
		1 × 10^8^	231.797
		1 × 10^9^	236.3918
		1 × 10^10^	152.3746
Larvae	*Trichoderma atroviride*	1 × 10^6^	255.8789
		1 × 10^7^	245.312
		1 × 10^8^	229.9565
		1 × 10^9^	215.3878
		1 × 10^10^	207.9787

In most of the study groups, the LT_50_ value decreased with an increase in concentration. The adult populations were killed faster than the larval populations (**Table 2**).

### 3.7 Confirmation of the pathogenic activity of the fungi

**Figure 4:**
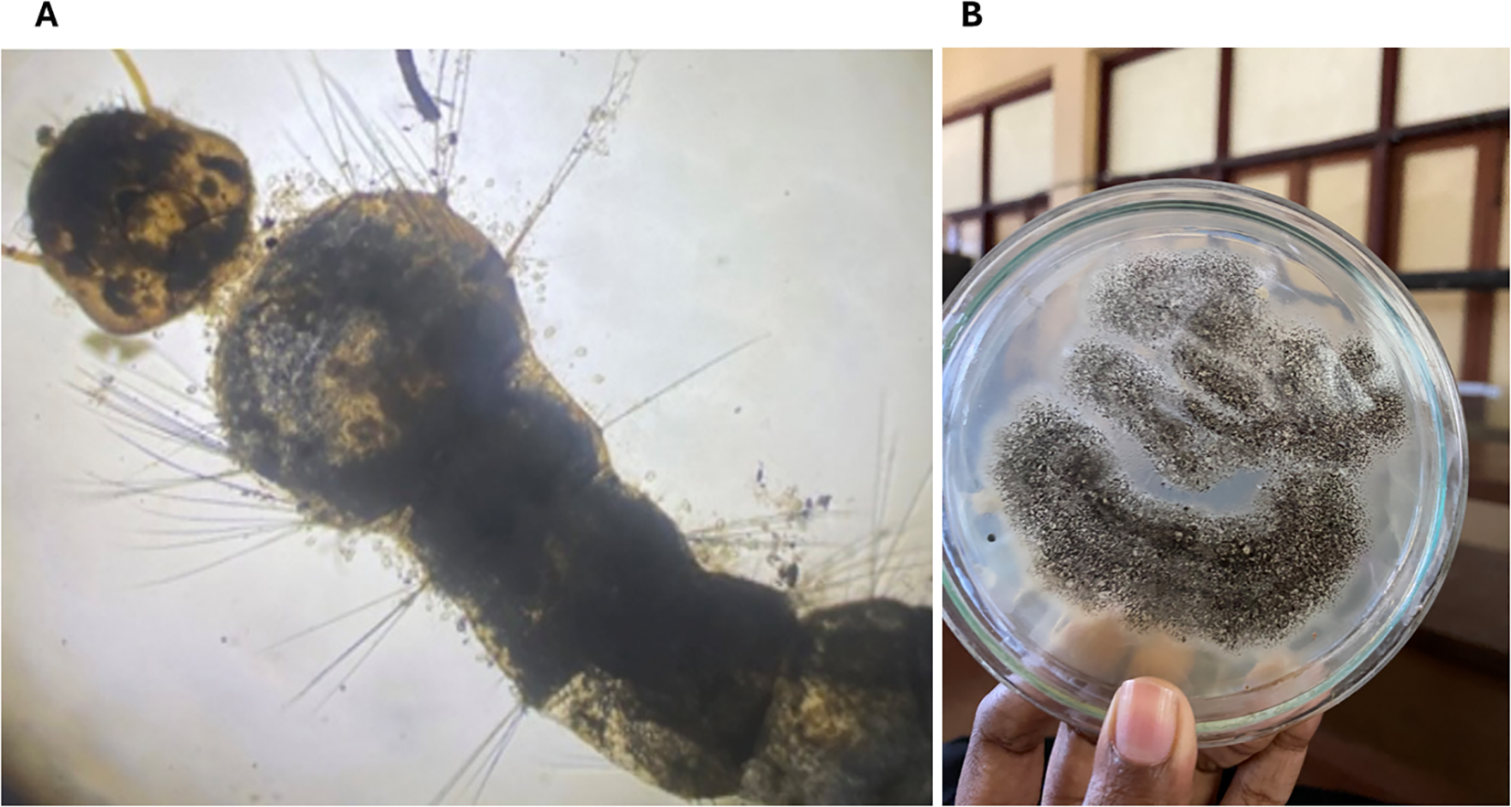
*Aedes aegypti* mosquitoes exposed to EPF indicating pathogenesis. **(A)** *Aedes aegypti* larvae exposed to *Trichoderma atroviridae* exhibiting the growth of fungal conidia; **(B)** *Aspergillus niger* re-isolated from a dead adult mosquito after the bioassay.

A typical fungal infection was re-confirmed in dead mosquito larvae and adults from the bioassay experiments by culturing in a PDA medium over time (**Figure 4**).

## 4 Discussion

The development of resistance to commonly used synthetic insecticides and the exponential increase in dengue incidences worldwide have highlighted the urgent need for new mosquito control methods. Entomopathogenic fungi are underexplored and underutilized as mosquito control agents. In the present study, we examined the potential use of *A. niger* and *T. atroviride*, respectively, as control agents for *Ae. aegypti*. We evaluated the efficacy of *A*. *niger* and *T*. *atroviride* spores against the larval, pupal, and adult stages of *Ae. aegypti* mosquitoes belonging to pyrethroid-resistant and pyrethroid-susceptible populations.

The findings of this study highlight the significant larvicidal potential of *A. niger* and *T. atroviride*, independent of the development of resistance to synthetic insecticides in mosquitoes. As the mosquito population in Sri Lanka has developed a high level of resistance to pyrethroid insecticides, our study may provide an eco-friendly larval control method. Many studies done in different parts of the world have highlighted the potential of fungi in terms of versatility. *Beauveria bassiana* and *M. anisopliae* fungal species have shown a significant reduction in both the larvae and adults *of Ae. aegypti* mosquitoes. A study done in Brazil has resulted in an approximate 80% reduction in larval populations tested for *M. anisopliae* (Carolino et al., 2014; Quinelato et al., 2012). Similarly, in Mexico, the use of *B. bassiana* has resulted in more than a 90% death rate among adult mosquitoes (García-Munguía et al., 2011). Laboratory trials carried out for *M. anisopliae* in Brazil, where dengue is emerging, have resulted in high larval mortalities (Bitencourt et al., 2023). A field experiment carried out in India has shown a significant reduction in mosquito densities when treated with *B. bassiana* (Veerwal et al., 2022). Miranpuri and Khachatourians (1990) demonstrated that *Ae. aegypti* larvae treated with *B. bassiana* conidia showed 50% mortality. However, our findings suggest that both *A*. *niger* and *T*. *atroviride* larvae show mortality of less than 50% at 96 h post-exposure for both *A*. *niger* and *T*. *atroviride* showing low efficacy, compared to *B. bassiana* or *M. anisopliae.* Furthermore, Soni and Prakash (2013) revealed that after an hour of treatment, the second instar larvae showed zero mortality in responding to the silver nanoparticles synthesized using *A*. *niger*. Similarly, the current findings of larvicidal bioassays conducted using *A*. *niger* spores suggest that zero mortality occurs at an hour of exposure, regardless of the type of population. However, a study conducted by Awad et al. (2022) revealed 100% mortality in both larval and pupal populations when exposed to silver nanoparticles synthesized by the metabolites of *A. niger* indicating high toxicity. Perera et al. (2023) studied the potential of metabolic extracts of *T. longibrachiatum* and *T. viride* against *Ae*. *aegypti* and *Ae. albopictus*. The study revealed high insecticidal activity against both mosquito vectors. Thus, further experiments are needed to confirm the role played by the metabolites excreted by the fungi.

Several factors have been suggested to be responsible for larval mortality due to EPF. Lacey et al. (1988) reported that larval mortality may occur due to blockage of the trachea and siphon of the mosquito larvae by the fungus. Researchers have also observed the presence of propagules in the larval gut and peri-spiracular lobes (Miranpuri and Khachatourians, 1990). Bhatt et al. (2013) suggested that the larvicidal effect was due to multiple factors, including asphyxiation and stress-induced apoptosis by spore-bound proteases. In contrast to these observations, blastospores on the larval surface and gut indicate larval penetration (Alkhaibari et al., 2016). Goettel (1988) hypothesized that variations in larval mortality during the bioassay might be caused by the inability of the ingested conidia to be digested and expelled in a viable form, which may influence the low mortality rates after some time. Overall, both fungal species in this study showed variations in larval mortality at each concentration and even among the replicates of the same concentration. Clark et al. (1968) also suggested that conidial dust from various strains of *B. bassiana* may result in varied mortality rates in mosquito larvae of several *Culex* sp. but not *Aedes.* They clarified that the longer time needed for a fungal infection to form in the larval peri-spiracular lobes was the cause of the observed variation in susceptibility. In the present study, Tukey’s pairwise comparisons of cumulative larval mortalities against time of exposure reveal significant differences in mortality with respect to time.

When considering the LC_50_ and LC_90_ values, there is no significant difference between the efficacy of *A*. *niger* and *T*. *atroviride* (*p* = 0.1824). A similar scenario can be observed with LT_50_ values as they are lower in *A. niger* than in *T. atroviride.* While there is a substantial difference in the mean values of LT_50_, the statistical analysis reveals that the significance is marginal but not conclusive (*p* = 0.05423). An effective larvicide may require rapid action potential in killing the larval stage; as time goes on, the larval stage might transform into pupae as a counteraction of withstanding unfavorable conditions. Hence, considering all these factors, *A*. *niger* can be more competent than *T*. *atroviride* as a larvicide.

It was of interest to observe that in both mosquito colonies, larval mortality did not reach 100% in response to either fungi strain. It has been suggested that mosquito immune response stimulated due to fungi may be responsible for this effect (Tawidian et al., 2019). A set of receptors found on the mosquito cell surface is able to detect fungal molecules or secondary metabolites secreted by EPF, which stimulate different immune signaling pathways in the mosquito such as Toll, immune deficiency, and Janus kinase/signal transducer and activator of transcription (Cherry and Silverman, 2006; Dimopoulos, 2003). Further studies are needed to identify the responsible immune signaling pathways for lower larval mortality.

Renuka et al. (2023) reported a delayed pupation when *Ae. aegypti* larvae were treated with the fungal conidia of *B. bassiana*. In a study on the late effect of *B. bassiana* on the larval stages of *Ae. aegypti*, it was observed that it took 36 days for the larvae to complete the larval stage upon exposure to *B. bassiana* (Quintero-Zapata et al., 2022). Current findings exhibit regular duration for pupation in the USJ population. However, the USJ and NeO populations treated with *A. niger* took 9 days to onset pupation. It has been suggested that developmental delays could be observed with mosquito larvae exposed to fungal entomopathogens. Several studies have reported scenarios similar to *Ae. albopictus*, *Cx. pipiens*, and *Anopheles gambiae* larvae exposed to various EPF, including *B. bassiana* and *M. ansiopliae* (Shoukat et al., 2016, 2020). The delayed pupation could be attributed to their natural life cycles being affected. A similar scenario was observed in several moth species exposed to *B. bassiana*; *M. anisopliae* have been attributed to metabolic changes and reduced larvae feeding resulting from fungal infection (Hussain et al., 2009). This delay may lead to higher population doubling time, impacting the disease transmission potential of the mosquito vector (Renuka et al., 2023). However, the population treated with the 1 × 10^10^ conidia/ml concentration of *T. atroviride* shows an unusually higher rate of pupation, even higher than the negative control, on the 8th and 9th days. The underlying cause for this scenario is currently unknown.

No pupal mortality was observed in any of the assays for either pyrethroid-resistant or pyrethroid-susceptible populations, confirming the absence of pupicidal activity of both *A. niger* and *T. atroviride*. However, unlike the larval stage, the type of population significantly influenced pupal emergence for both fungal species. Pupal emergence also varied significantly with different concentrations within the same fungal species. These differences, combined with the lack of pupal mortality, may stem from the inability of larvae to digest the ingested conidia, which are expelled in a viable form, potentially affecting their pupation process. Overall, *T. atroviride* had a minimal impact (*p* = 0.00054) on pupation compared to *A. niger*, showing trends similar to the negative controls.

Current findings suggest that both *A. niger* and *T. atroviride* show rather slow but gradually increasing mortality rates upon increasing spore concentrations in adults of *Ae. aegypti* of both USJ and NeO populations. According to Scholte et al. (2007), in *Ae. aegypti* mosquitoes, a higher spore dosage was linked to a higher mortality rate, implying that the mosquitoes’ immune system is only partially efficient in fighting fungal infection at low concentrations. It becomes less effective at higher conidial doses. Re-isolation of the fungi from the dead adult mosquito body suggests entomopathogenic activity of the two fungi species used.

Adult mosquitoes are the primary vectors of dengue. Therefore, control of the adult *Ae. aegypti* is crucial. Upon exposure to fungal spores of both *A. niger* and *T. atroviride*, adult mortality has reached 100% at all concentrations regardless of the type of population. This reveals that the prior susceptible/resistant state of a mosquito population does not affect the entomopathogenicity of the fungi tested. Hence, it can be stated that the spores of both *A. niger* and *T. atroviride* are successful tools for controlling the primary vector stage of *Ae. aegypti*.

A study in which *M. anisopliae* was tested on *Ae. aegypti*, LT_50_ of mosquitoes infected with fungus varied from 3.1 ± 0.2 days for male *Ae. aegypti* and 4.1 ± 0.3 days for female *Ae. aegypti* (Scholte et al., 2007). The present study reveals similar LT_50_ values (LT_50_ is approximately 4 to 5 days at 1 × 10^9^ conidia/ml concentration) for both *A. niger* and *T. atroviride.* At all concentrations, LT_50_ values shown by *A. niger* are significantly lower (*p* = 0.00275) lower than that of *T. atroviride*. It suggests that as a potential bioinsecticide against *Ae. aegypti* adult mosquitoes, *A. niger* is more competent than *T. atroviride.*

Based on current findings, the effects of *A. niger* and *T. atroviride* on both larval and adult stages and also on pupal emergence rates make both fungal species competent in the control of *Ae. aegypti.* However, the method of delivering the EPF to the target stage is important. The persistence and virulence of spores are key factors that need to be considered in the method of exposure or delivery.

According to Islam et al. (2021), there are several benefits of EPF over synthetic insecticides for mosquito control. These include being host-specific and environment-friendly, with a unique mode of action that can develop low or no resistance, etc. However, there are limitations as well. Germination and infection require a certain amount of time without the use of fungicides, as well as ideal climatic factors (high relative humidity and comfortable temperature). They are typically expensive and challenging to create on a commercial scale. Entomopathogenic fungi have a relatively short shelf life, it takes 2 to 3 weeks to control insects. Secondary metabolites produced by fungi, which can be toxins, pose a threat to the ecosystem and biodiversity. This is mainly why EPF is applied as formulations.

In Sri Lanka, EPF is heavily understudied, and only a few studies have reported the efficiency of EPF that can be used as potential bioinsecticides for *Ae. aegypti* mosquito control. The findings of this study can be improved to explore optimal conidial doses, mass culturing of fungi, the optimal shelf life of fungi and their conidia, and optimal suspension for the conidia and also to explore combined formulations such as nanoparticles and oil formulations.

## 5 Conclusion

This is a pioneer preliminary study in Sri Lanka on the evaluation of mycoparasitic *T. atroviride* and an EPF *A. niger* against the dengue vector, *Ae. aegypti*. In this study, it was observed that both *A. niger* and *T. atroviride* were partially effective as larvicides against *Ae. aegypti*. A proportion of larvae remained resistant in both instances. Both fungal species delayed the development of larvae into pupae, but the effect was not pupicidal. Both *A. niger* and *T. atroviride* were found to be fully effective as adulticides for *Ae. aegypti*. There was no significant difference among the pyrethroid-susceptible or pyrethroid-resistant mosquito populations, suggesting that prior development of resistance will not affect the entomopathogenic activity. Therefore, it can be concluded that at a preliminary level, both *A. niger* and *T. atroviride* can be effective as potential bioinsecticides to control both larval and adult stages of *Ae. aegypti* mosquito populations.

## Data Availability

The datasets presented in this study can be found in online repositories. The names of the repository/repositories and accession number(s) can be found in the article/**Supplementary Material**.

## Supplementary material

Supplementary Figure 1 Kruskal Wallis multiple comparisons for larval mortality among USJ population for spores of *A. niger* for different time **(A)** intervals and concentrations **(B)**.

Supplementary Figure 2 Kruskal Wallis multiple comparisons for larval mortality among NO population for spores of *A. niger* for different time **(A)** intervals and concentrations **(B)**.

Supplementary Figure 3 Kruskal Wallis multiple comparisons for larval mortality among USJ population for spores of *T. atroviride* for different time **(A)** intervals and concentrations **(B)**.

Supplementary Figure 4 Kruskal Wallis multiple comparisons for larval mortality among NO population for spores of *T. atroviride* for different time **(A)** intervals and concentrations **(B)**.

Supplementary Figure 5 Kruskal Wallis multiple comparisons for pupal emergence among USJ and NO populations for spores of *A. niger* and *T. atroviride* for different time intervals. **(A)** *A. niger* on USJ population, **(B)** *A. niger* on NO population, **(C)** *T. atroviride* on USJ population, **(D)** *T. atroviride* on NO population.

Supplementary Figure 6 Kruskal Wallis multiple comparisons for pupal emergence among USJ and NO populations for spores of *A. niger* and *T. atroviride* for different concentrations. **(A)** *A. niger* on USJ population, **(B)** *A. niger* on NO population, **(C)** *T. atroviride* on USJ population, **(D)** *T. atroviride* on NO population.

Supplementary Figure 7 Kruskal Wallis multiple comparisons for adult mortality among USJ and NO populations for spores of *A. niger* and *T. atroviride* for different time intervals. **(A)** *A. niger* on USJ population, **(B)** *A. niger* on NO population, **(C)** *T. atroviride* on USJ population, **(D)** *T. atroviride* on NO population.

Supplementary Figure 8 Kruskal Wallis multiple comparisons for adult mortality among USJ and NO populations for spores of *A. niger* and *T. atroviride* for different concentrations. **(A)** *A. niger* on USJ population, **(B)** *A. niger* on NO population, **(C)** *T. atroviride* on USJ population, **(D)** *T. atroviride* on NO population.

## References

[B1] Accoti A. Engdahl C. S. Dimopoulos G. (2021). Discovery of novel entomopathogenic fungi for mosquito-borne disease control. Front. Fungal Biol. 2. doi: 10.3389/ffunb.2021.637234 PMC10512396 37744144

[B2] Achee N. L. Grieco J. P. Vatandoost H. Seixas G. Pinto J. Ching-Ng L.. (2019). Alternative strategies for mosquito-borne arbovirus control. PloS Negl. Trop. Dis. 13, e0007275. doi: 10.1371/journal.pntd.0007275 30605475 PMC6317787

[B3] Alkhaibari A. M. Carolino A. T. Yavasoglu S. I. Maffeis T. Mattoso T. C. Bull J. C.. (2016). *Metarhizium brunneum* blastospore pathogenesis in *Aedes aegypti* larvae: attack on several fronts accelerates mortality. PloS Pathogens 12, e1005715. doi: 10.1371/journal.ppat.1005715 27389584 PMC4936676

[B4] Awad M. A. Eid A. M. Elsheikh T. M. Al-Faifi Z. E. Saad N. Sultan M. H.. (2022). Mycosynthesis, characterization, and mosquitocidal activity of silver nanoparticles fabricated by *Aspergillus niger* strain. J. Fungi 8, 396. doi: 10.3390/jof8040396 PMC9026153 35448627

[B5] Bhatt S. Gething P. W. Brady O. J. Messina J. P. Farlow A. W. Moyes C. L.. (2013). The global distribution and burden of dengue. Nature 496, 504–507. doi: 10.1038/nature12060 23563266 PMC3651993

[B6] Bitencourt R. D. O. B. Santos-Mallet J. R. D. Lowenberger C. Ventura A. Gôlo P. S. Bittencourt V. R. E. P.. (2023). A novel model of pathogenesis of *Metarhizium anisopliae* propagules through the midguts of *Aedes aegypti* larvae. Insects 14, 328. doi: 10.3390/insects14040328 37103143 PMC10146130

[B7] Brivio M. F. Mastore M. (2020). When appearance misleads: The role of the entomopathogen surface in the relationship with its host. Insects 11, 387. doi: 10.3390/insects11060387 32585858 PMC7348879

[B8] Carolino A. T. Paula A. R. Silva C. P. Butt T. M. Samuels R. I. (2014). Monitoring persistence of the entomopathogenic fungus *Metarhizium anisopliae* under simulated field conditions with the aim of controlling adult *Aedes aegypti* (Diptera: Culicidae). Parasitol. Vectors 7, 198. doi: 10.1186/1756-3305-7-198 PMC4021620 24766705

[B9] Chen S. Cao Z. Jiao L. Chen W. Prettner K. Kuhn M.. (2024). The global economic burden of dengue in 2020–2050: estimates and projections for 141 countries and territories. Lancet 16, 935–941. doi: 10.2139/ssrn.4691773

[B10] Cherry S. Silverman N. (2006). Host-pathogen interactions in drosophila: new tricks from an old friend. Nat. Immunol. 7, 911–917. doi: 10.1038/ni1388 16924255

[B11] Clark T. B. Kellen R. K. Fukuda T. Lindegren J. E. (1968). Field and laboratory studies on the pathogenicity of the fungus *Beuuveria bassiunuto* three genera of mosquitoes. J. Invertebr. Pathol. 11, 1–7. doi: 10.1016/0022-2011(68)90047-5 5654770

[B12] Diba K. Kordbacheh P. Mirhendi S. H. Rezaie S. Mahmoudi M. (2007). Identification of *Aspergillus* species using morphological characteristics. Pak. J. Med. Sci. 23, 867–872.

[B13] Dimopoulos G. (2003). Insect immunity and its implication in mosquito-malaria interactions. Cell. Microbiol. 5, 3–14. doi: 10.1046/j.1462-5822.2003.00252.x 12542466

[B14] Facchinelli L. Badolo A. McCall P. J. (2023). Biology and behaviour of *Aedes aegypti* in the human environment: Opportunities for vector control of arbovirus transmission. Viruses 15, 636. doi: 10.3390/v15030636 36992346 PMC10053764

[B15] Fernando H. S. D. Saavedra-Rodriguez K. Perera R. Black W. C. De Silva B.G.D.N.K. (2020). Resistance to commonly used insecticides and underlying mechanisms of resistance in *Aedes aegypti* (L.) from Sri Lanka. Parasitol. Vectors 13, 407. doi: 10.1186/s13071-020-04284-y PMC7418196 32778147

[B16] Fernando S. D. Hapugoda M. Perera R. Saavedra-Rodriguez K. Black W. C. De Silva N. K. (2018). First report of V1016G and S989P knockdown resistant (kdr) mutations in pyrethroid-resistant Sri Lankan *Aedes aegypti* mosquitoes. Parasitol. Vectors 11, 526. doi: 10.1186/s13071-018-3113-0s PMC6158842 30257701

[B17] García-Munguía A. M. Garza-Hernández J. A. Rebollar-Tellez E. A. Rodríguez-Pérez M. A. Reyes-Villanueva F. (2011). Transmission of *Beauveria bassiana* from male to female *Aedes aegypti* mosquitoes. Parasitol. Vectors 4, 24. doi: 10.1186/1756-3305-4-24 PMC3051917 21352560

[B18] Goettel M. S. (1988). Pathogenesis of the hyphomycete *Tolypocladium cylindrosporum* in the mosquito *Aedes aegypti* . J. Invertebr. Pathol. 51, 259–274. doi: 10.1016/0022-2011(88)90033-X 3373005

[B19] Hussain A. Tian M. Y. He Y. R. Ahmed S. (2009). Entomopathogenic fungi disturbed the larval growth and feeding performance of *Ocinara varians* (Lepidoptera: Bombycidae) larvae. Insect Sci. 16, 511–517. doi: 10.1111/j.1744-7917.2009.01272.x

[B20] Islam W. Adnan M. Shabbir A. Naveed H. Abubakar Y. S. Qasim M.. (2021). Insect-fungal-interactions: A detailed review on entomopathogenic fungi pathogenicity to combat insect pests. Microb. Pathog. 159, 105122. doi: 10.1016/j.micpath.2021.105122 34352375

[B21] Isman M. B. (2020). Botanical insecticides in the twenty-first century—fulfilling their promise? Annu. Rev. Entomol. 65, 233–249. doi: 10.1146/annurev-ento-011019-025010 31594414

[B22] Konara U. A. Thambugala K. M. Karunarathna S. C. Ediriweera A. Hapuarachchi K. K. (2024). Unveiling the hidden diversity of *Ganoderma* (Ganodermataceae, Polyporales) in Sri Lanka: the first report of *G. angustisporum*, *G. ellipsoideum*, and *G. orbiforme* . N. Z. J. Bot., 1–25. doi: 10.1080/0028825X.2024.2415555

[B23] Lacey L. A. Grzywacz D. Shapiro-Ilan D. I. Frutos R. Brownbridge M. Goettel M. S. (2015). Insect pathogens as biological control agents: Back to the future. J. Invertebr. Pathol. 132, 1–41. doi: 10.1016/j.jip.2015.07.009 26225455

[B24] Lacey C. M. Lacey L. A. Roberts D. R. (1988). Route of invasion and histopathology of *Metarhizium anisopliae* in *Culex quinquefasciatus* . J. Invertebr. Pathol. 52, 108–118. doi: 10.1016/0022-2011(88)90109-7 3418133

[B25] Mascarin G. M. Lopes R. B. Delalibera Í. Fernandes É.K.K. Luz C. Faria M. (2019). Current status and perspectives of fungal entomopathogens used for microbial control of arthropod pests in Brazil. J. Invertebr. Pathol. 165, 46–53. doi: 10.1016/j.jip.2018.01.001 29339191

[B26] Meghani Z. Boëte C. (2018). Genetically engineered mosquitoes, Zika and other arboviruses, community engagement, costs, and patents: Ethical issues. PloS Negl. Trop. Dis. 12, e0006501. doi: 10.1371/journal.pntd.0006501 30048441 PMC6062015

[B27] Mendis B. A. N. Peiris V. Harshani W. A. K. Fernando H. S. D. De Silva B.G.D.N.K. (2024). Fine-scale monitoring of insecticide resistance in *Aedes aegypti* (Diptera: Culicidae) from Sri Lanka and modeling the phenotypic resistance using rational approximation. Parasitol. Vectors 17, 18. doi: 10.1186/s13071-023-06100-9 PMC10785423 38216956

[B28] Miranpuri G. S. Khachatourians G. G. (1990). Larvicidal activity of blastospores and conidiospores of *Beauveria bassiana* (strain GK 2016) against age groups of *Aedes aegypti* . Vet. Parasitol. 37, 155–162. doi: 10.1016/0304-4017(90)90070-R 2251749

[B29] Paz-Bailey G. Adams L. E. Deen J. Anderson K. B. Katzelnick L. C. (2024). Dengue. Lancet 403, 667–682. doi: 10.1016/S0140-6736(23)02576-X 38280388 PMC12372472

[B30] Perera D. S. Tharaka W. H. Amarasinghe D. Wickramarachchi S. R. (2023). Extracellular extracts of antagonistic fungi, *Trichoderma longibrachiatum* and *Trichoderma viride*, as larvicides against dengue vectors, *Aedes aegypti* and *Aedes albopictus* . Acta Trop. 238, 106747. doi: 10.1016/j.actatropica.2022.106747 36368414

[B31] Qin Y. Liu X. Peng G. Xia Y. Cao Y. (2023). Recent advancements in pathogenic mechanisms, applications and strategies for entomopathogenic fungi in mosquito biocontrol. J. Fungi 9, 746. doi: 10.3390/jof9070746 PMC10381795 37504734

[B32] Quinelato S. Golo P. S. Perinotto W. M. Sá F. A. Camargo M. G. Angelo I. C.. (2012). Virulence potential of *Metarhizium anisopliae* sl isolates on *Rhipicephalus* (*Boophilus*) *microplus* larvae. Vet. Parasitol. 190, 556–565. doi: 10.1016/j.vetpar.2012.06.028 22840642

[B33] Quintero-Zapata I. Flores-González M. S. Luna-Santillana E. J. Arroyo-González N. Gandarilla-Pacheco F. L. (2022). Late effects of *Beauveria bassiana* on larval stages of *Aedes aegypti* Linneo 1762 (Diptera: Culicidae). Braz. J. Biol. 82, e237789. doi: 10.1590/1519-6984.237789 33978078

[B34] R Development Core Team (2024). R: A language and environment for statistical computing. Version 4.3.3 [software] (Vienna, Austria: R Foundation for Statistical Computing). Available at: http://www.r-project.org/ (Accessed February 25, 2024).

[B35] Renuka S. Vani H C. Alex E. (2023). Entomopathogenic fungi as a potential management tool for the control of urban malaria vector, *Anopheles stephensi* (Diptera: Culicidae). J. Fungi 9, 223. doi: 10.3390/jof9020223 PMC9966075 36836337

[B36] Rodrigues-Alves M. L. Melo-Júnior O. A. D. O. Silveira P. Mariano R. M. D. S. Leite J. C. Santos T. A. P.. (2020). Historical perspective and biotechnological trends to block arboviruses transmission by controlling *Aedes aegypti* mosquitos using different approaches. Front. Med. 7. doi: 10.3389/fmed.2020.00275 PMC7325419 32656216

[B37] Rueda L. M. (2004). Pictorial keys for the identification of mosquitoes (Diptera: Culicidae) associated with dengue virus transmission (Zootaxa 589.Auckland: Magnolia Press).

[B38] Scholte E. J. Njiru B. N. Smallegange R. C. Takken W. Knols B. G. (2003). Infection of malaria (*Anopheles Gambiae* s.s.) and filariasis (*Culex quinquefasciatus*) vectors with the entomopathogenic fungus *Metarhizium anisopliae* . Malar. J. 2, 29. doi: 10.1186/1475-2875-2-29 14565851 PMC222926

[B39] Scholte E. J. Takken W. Knols B. G. J. (2007). Infection of adult *Aedes aegypti* and *Ae. albopictus* mosquitoes with the entomopathogenic fungus *Metarhizium anisopliae* . Acta Trop. 102, 151–158. doi: 10.1016/j.actatropica.2007.04.011 17544354

[B40] Sharma A. Sharma S. Yadav P. K. (2023). Entomopathogenic fungi and their relevance in sustainable agriculture: A review. Cogent. Food Agric. 9, 2180857. doi: 10.1080/23311932.2023.2180857

[B41] Shen D. Tang Z. Wang C. Wang J. Dong Y. Chen Y.. (2019). Infection mechanisms and putative effector repertoire of the mosquito pathogenic oomycete *Pythium guiyangense* uncovered by genomic analysis. PloS Genet. 15, e1008116. doi: 10.1371/journal.pgen.1008116 31017897 PMC6502433

[B42] Shoukat R. F. Freed S. Ahmad K. W. (2016). Evaluation of binary mixtures of entomogenous fungi and botanicals on biological parameters of *Culex pipiens* (Diptera: Culicidae) under laboratory and field conditions. Int. J. Mosq. Res. 3, 17–24.

[B43] Shoukat R. F. Zafar J. Shakeel M. Zhang Y. Freed S. Xu X.. (2020). Assessment of lethal, sublethal, and transgenerational effects of *Beauveria bassiana* on the demography of *Aedes albopictus* (Culicidae: Diptera). Insects 11, 178. doi: 10.3390/insects11030178 32168886 PMC7143237

[B44] Sirisena P. D. N. N. Noordeen F. (2014). Evolution of dengue in Sri Lanka—changes in the virus, vector, and climate. Int. J. Infect. Dis. 19, 6–12. doi: 10.1016/j.ijid.2013.10.012 24334026

[B45] Soni N. Prakash S. (2013). Possible mosquito control by silver nanoparticles synthesized by soil fungus (*Aspergillus Niger* 2587). Adv. Nanopart. 2, 125–132. doi: 10.4236/anp.2013.22021

[B46] Souza-Neto J. A. Powell J. R. Bonizzoni M. (2019). *Aedes aegypti* vector competence studies: A review. Infect. Genet. Evol. 67, 191–209. doi: 10.1016/j.meegid.2018.11.009 30465912 PMC8135908

[B47] Tawidian P. Rhodes V. L. Michel K. (2019). Mosquito-fungus interactions and antifungal immunity. Insect Biochem. Mol. Biol. 111, 103182. doi: 10.1016/j.ibmb.2019.103182 PMC6639037 31265904

[B48] Tayal A. Kabra S. K. Lodha R. (2023). Management of dengue: an updated review. Indian J. Pediatr. 90, 168–177. doi: 10.1007/s12098-022-04394-8 36574088 PMC9793358

[B49] Thalagala N. Tissera H. Palihawadana P. Amarasinghe A. Ambagahawita A. Wilder-Smith A.. (2016). Costs of dengue control activities and hospitalizations in the public health sector during an epidemic year in urban Sri Lanka. PloS Negl. Trop. Dis. 10, e0004466. doi: 10.1371/journal.pntd.0004466 26910907 PMC4766086

[B50] Thambugala K. M. Hyde K. D. Tanaka K. Tian Q. Wanasinghe D. N. Ariyawansa H. A.. (2015). Towards a natural classification and backbone tree for *Lophiostomataceae*, *Floricolaceae*, and *Amorosiaceae fam. nov* . Fungal Divers. 74, 199–266. doi: 10.1007/s13225-015-0348-3

[B51] Thambugala K. M. Wanasinghe D. N. Phillips A. J. L. Camporesi E. Bulgakov T. S. Phukhamsakda C.. (2017). Mycosphere notes 1–50: grass (Poaceae) inhabiting Dothideomycetes. Mycosphere 8, 697–796. doi: 10.5943/mycosphere/8/4/13

[B52] Veerwal B. Prasad A. Intodia A. (2022). Development of fungal entomopathogen *Beauveria bassiana* (Balsamo) formulations for control of mosquito larvae in the field. Environ. Conserv. 28, 373–378. doi: 10.53550/EEC.2022.v28i01.056

[B53] Vivekanandhan P. Bedini S. Shivakumar M. S. (2020). Isolation and identification of entomopathogenic fungus from Eastern Ghats of South Indian forest soil and their efficacy as biopesticide for mosquito control. Parasitol. Int. 76, 102099. doi: 10.1016/j.parint.2020.102099 32169659

[B54] Wang G. H. Gamez S. Raban R. R. Marshall J. M. Alphey L. Li M.. (2021). Combating mosquito-borne diseases using genetic control technologies. Nat. Commun. 12, 4388. doi: 10.1038/s41467-021-24654-z 34282149 PMC8290041

[B55] White T. J. Bruns T. Lee S. Taylor J. (1990). “Amplification and direct sequencing of fungal ribosomal RNA genes for phylogenetics,” in PCR Protocols: A guide to methods and applications, vol. 18 . Eds. Innis M. A. Gelfand D. H. Sninsky J. J. White T. J. (Academic Press Inc, New York), 315–322.

[B56] Wilson A. L. Courtenay O. Kelly-Hope L. A. Scott T. W. Takken W. Torr S. J.. (2020). The importance of vector control for the control and elimination of vector-borne diseases. PloS Negl. Trop. Dis. 14, e0007831. doi: 10.1371/journal.pntd.0007831 31945061 PMC6964823

[B57] World Health Organization (WHO) (2022). Manual for monitoring insecticide resistance in mosquito vectors and selecting appropriate interventions (Organização Mundial da Saúde), 1–65. Available at: https://www.who.int/publications/i/item/9789240051089 (Accessed June 28, 2023).

[B58] World Health Organization (WHO) (2024). Dengue and severe dengue. Available online at: https://www.who.int/news-room/fact-sheets/detail/dengue-and-severe-dengue (Accessed March 4, 2024).

[B59] Yapa A. T. Thambugala K. M. Samarakoon M. C. de Silva N. (2024). *Metarhizium* species as bioinsecticides: potential, progress, applications & future perspectives. N. Z. J. Bot. 63 (2–3), 1–23. doi: 10.1080/0028825X.2024.2325006

