# Supplementary figures and images for: Evaluation of mycoparasitic Trichoderma atroviride and entomopathogenic Aspergillus niger as potential bioinsecticides against the dengue vector, Aedes aegypti
Source: Front Cell Infect Microbiol. 2025 Apr 10;15:1502579. doi: 10.3389/fcimb.2025.1502579 (PMC12038353; doi:10.3389/fcimb.2025.1502579)

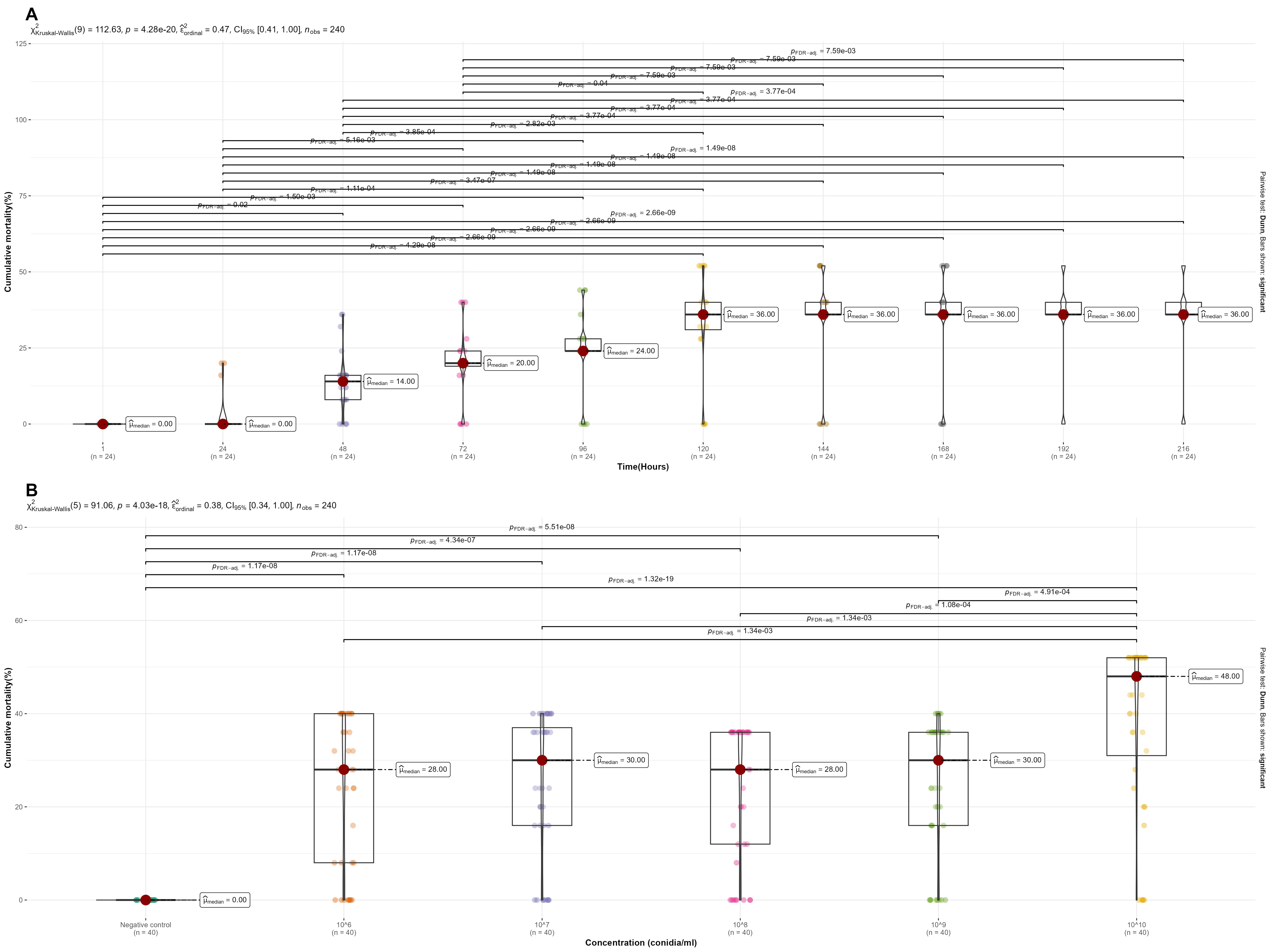

Supplement: Supplementary Figure 1 — Kruskal Wallis multiple comparisons for larval mortality among USJ population for spores of A. niger for different time (A) intervals and concentrations (B). [file Image1.jpeg]

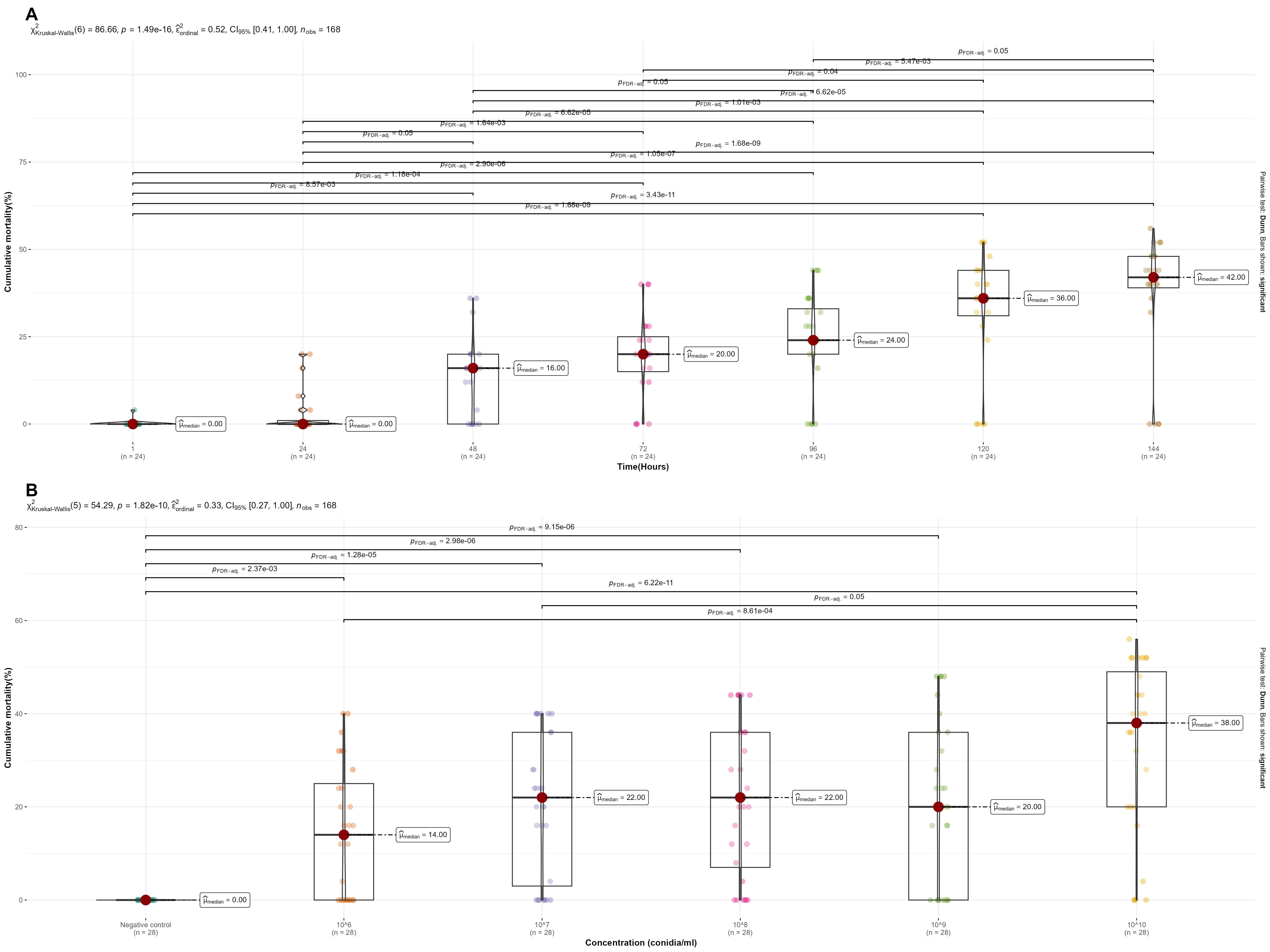

Supplement: Supplementary Figure 2 — Kruskal Wallis multiple comparisons for larval mortality among NO population for spores of A. niger for different time (A) intervals and concentrations (B). [file Image2.jpeg]

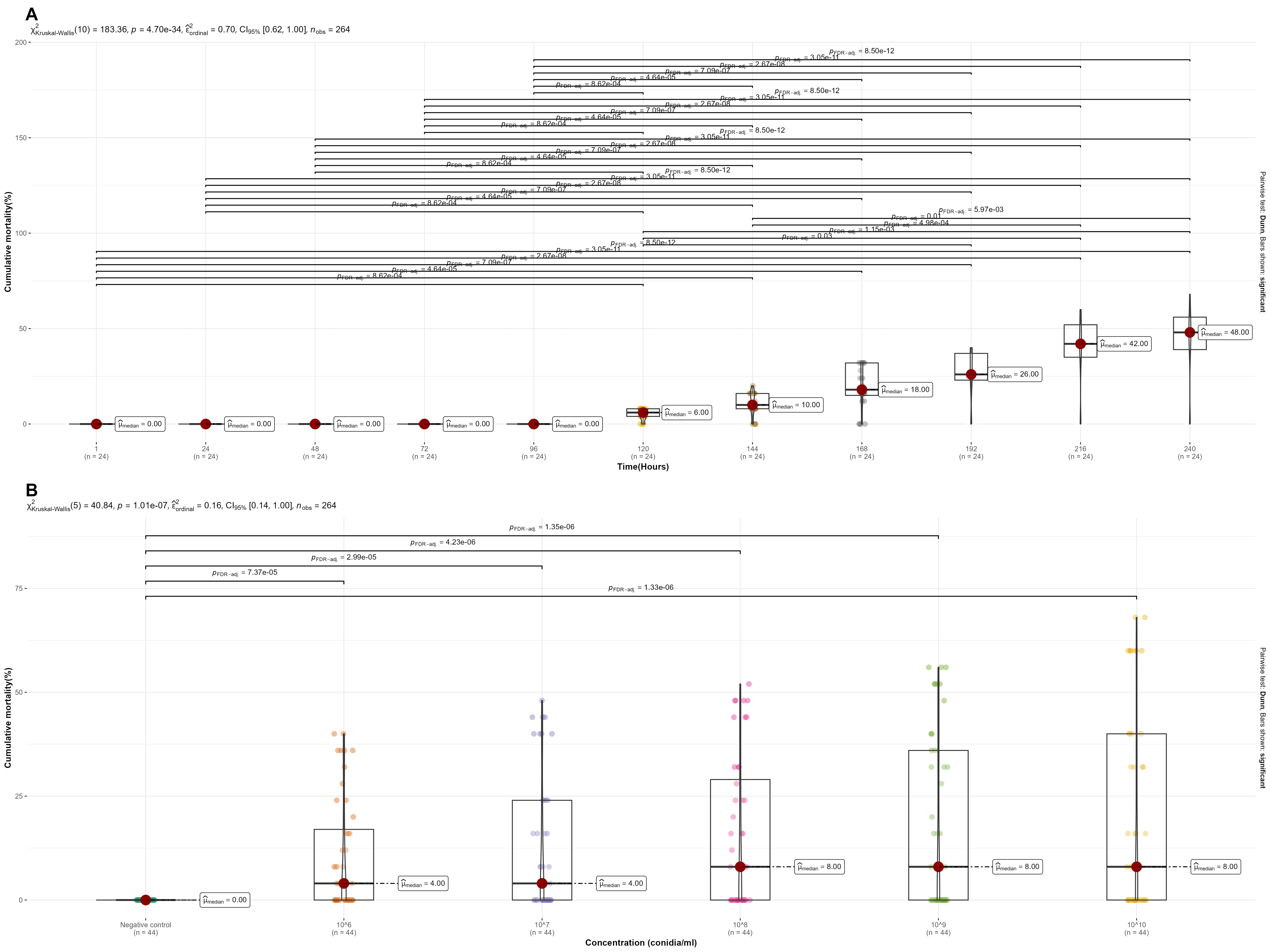

Supplement: Supplementary Figure 3 — Kruskal Wallis multiple comparisons for larval mortality among USJ population for spores of T. atroviride for different time (A) intervals and concentrations (B). [file Image3.jpeg]

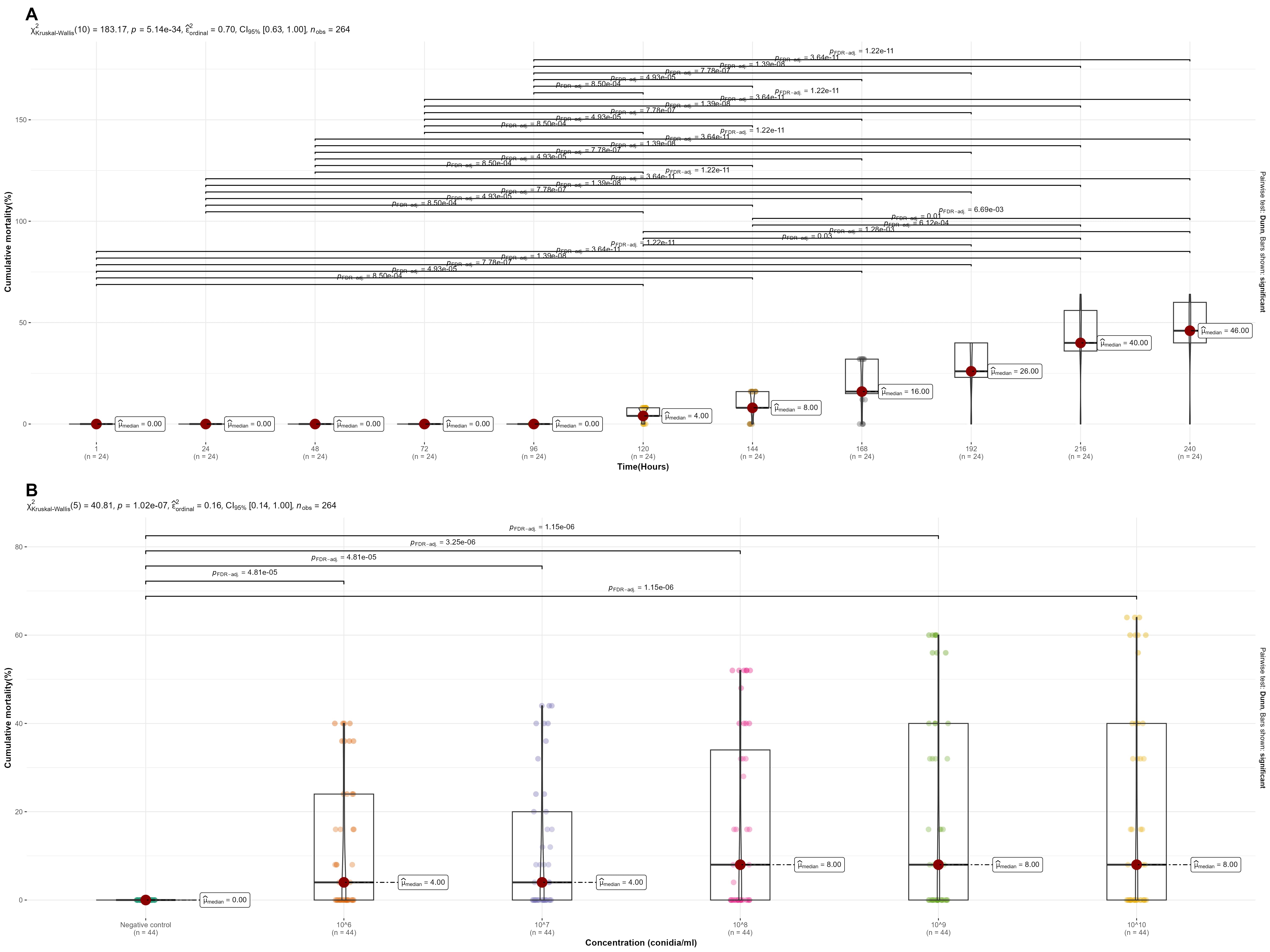

Supplement: Supplementary Figure 4 — Kruskal Wallis multiple comparisons for larval mortality among NO population for spores of T. atroviride for different time (A) intervals and concentrations (B). [file Image4.jpeg]

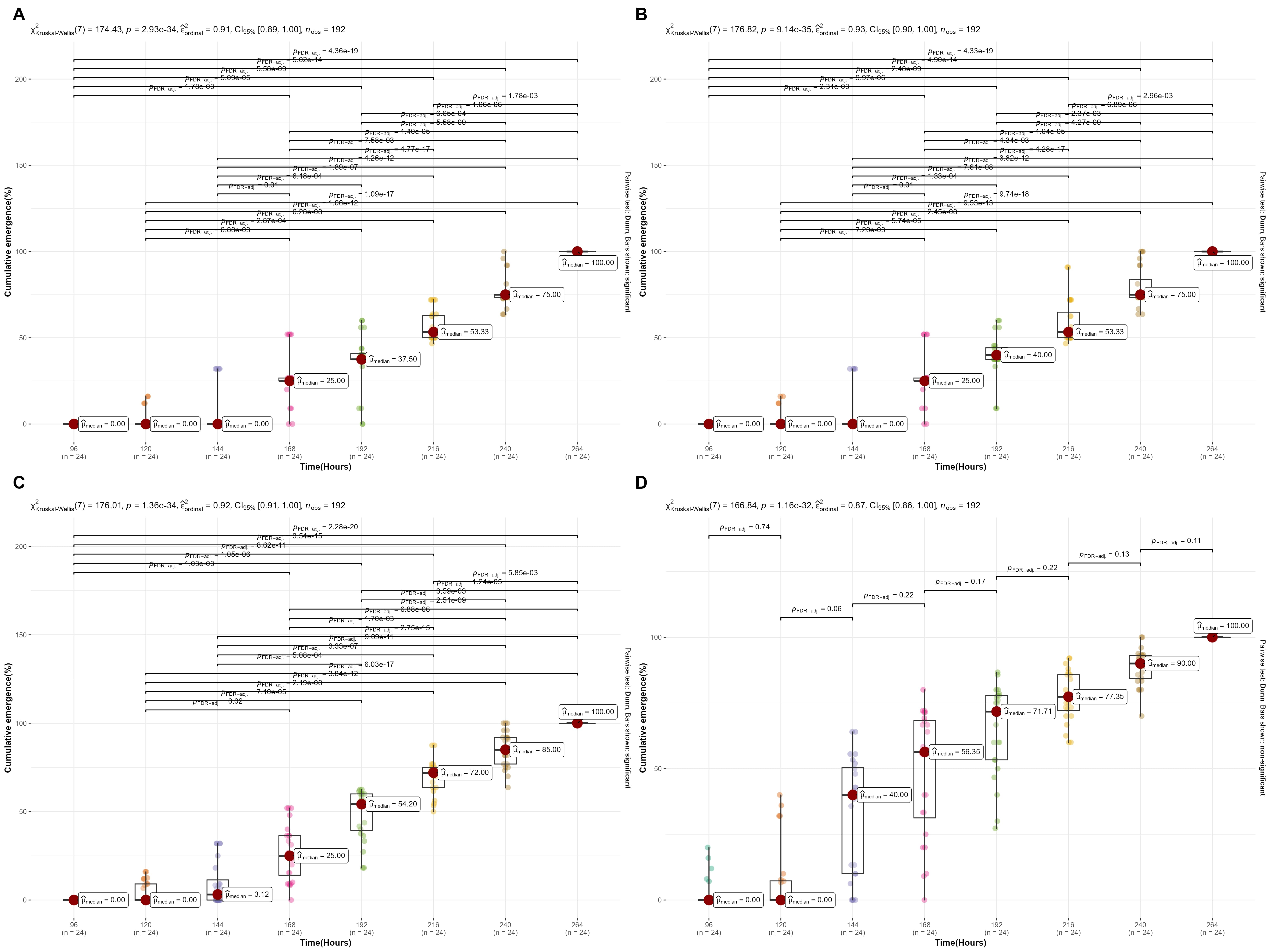

Supplement: Supplementary Figure 5 — Kruskal Wallis multiple comparisons for pupal emergence among USJ and NO populations for spores of A. niger and T. atroviride for different time intervals. (A) A. niger on USJ population, (B) A. niger on NO population, (C) T. atroviride on USJ population, (D) T. atroviride on NO population. [file Image5.jpeg]

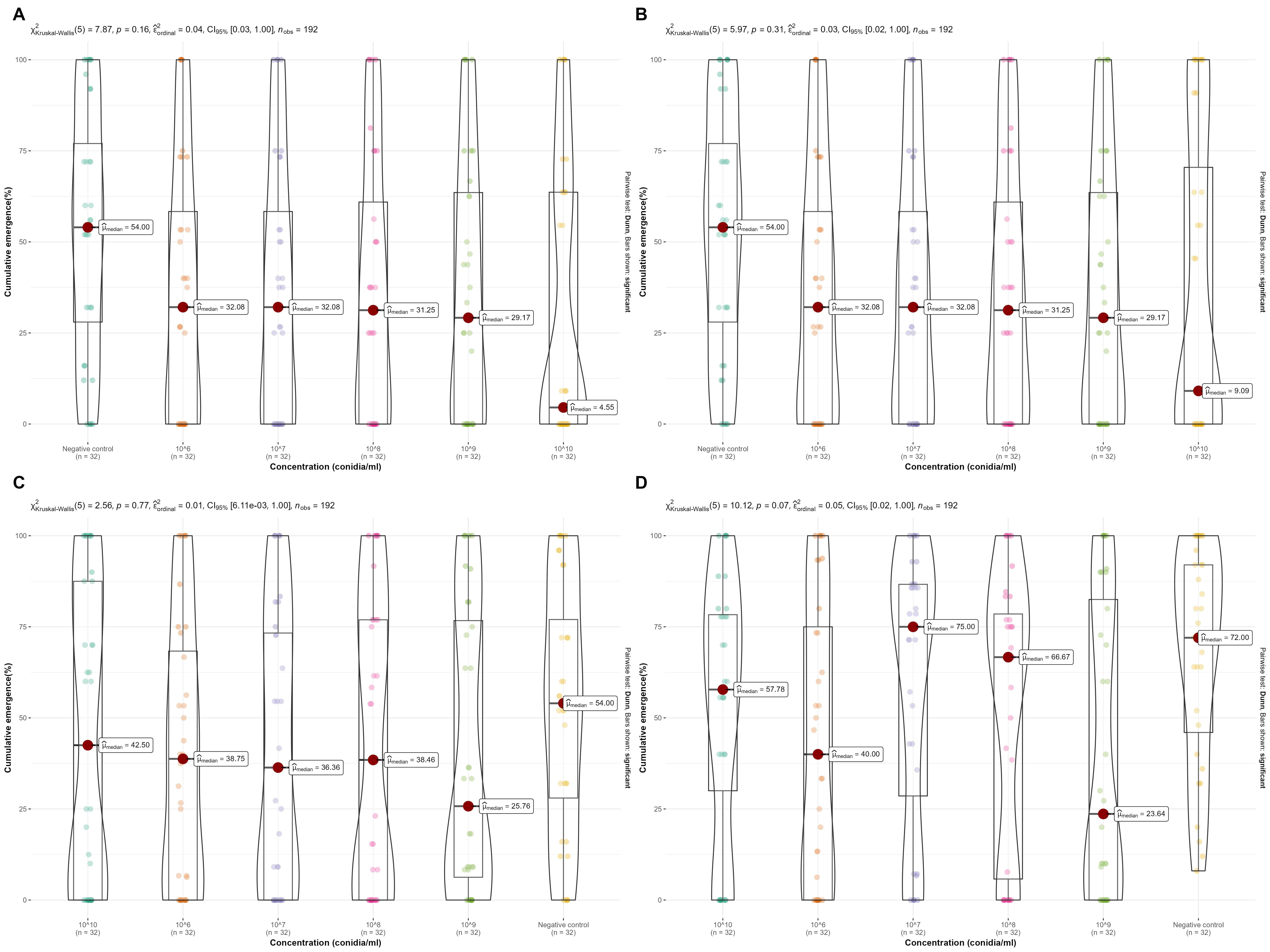

Supplement: Supplementary Figure 6 — Kruskal Wallis multiple comparisons for pupal emergence among USJ and NO populations for spores of A. niger and T. atroviride for different concentrations. (A) A. niger on USJ population, (B) A. niger on NO population, (C) T. atroviride on USJ population, (D) T. atroviride on NO population. [file Image6.jpeg]

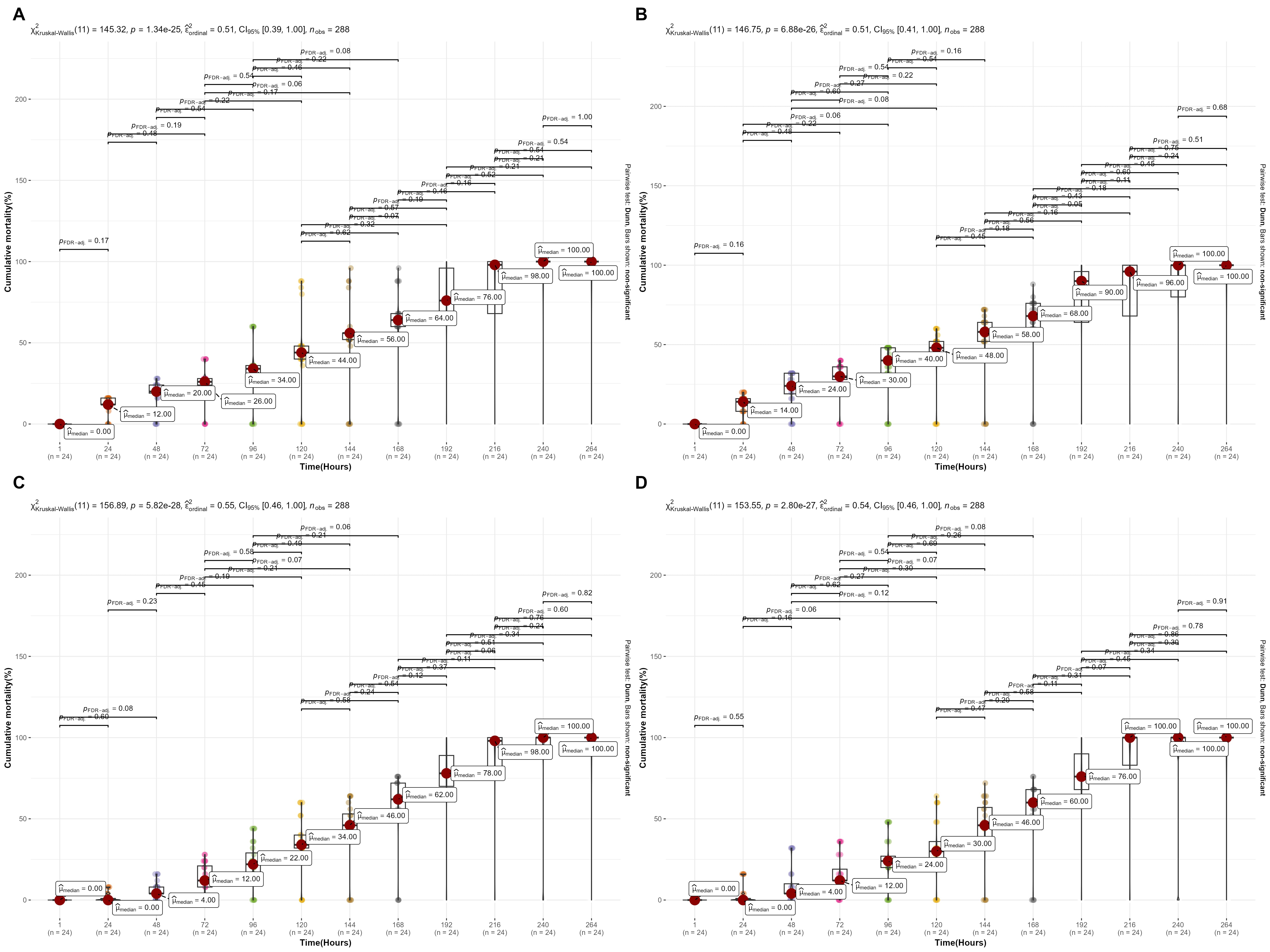

Supplement: Supplementary Figure 7 — Kruskal Wallis multiple comparisons for adult mortality among USJ and NO populations for spores of A. niger and T. atroviride for different time intervals. (A) A. niger on USJ population, (B) A. niger on NO population, (C) T. atroviride on USJ population, (D) T. atroviride on NO population. [file Image7.jpeg]

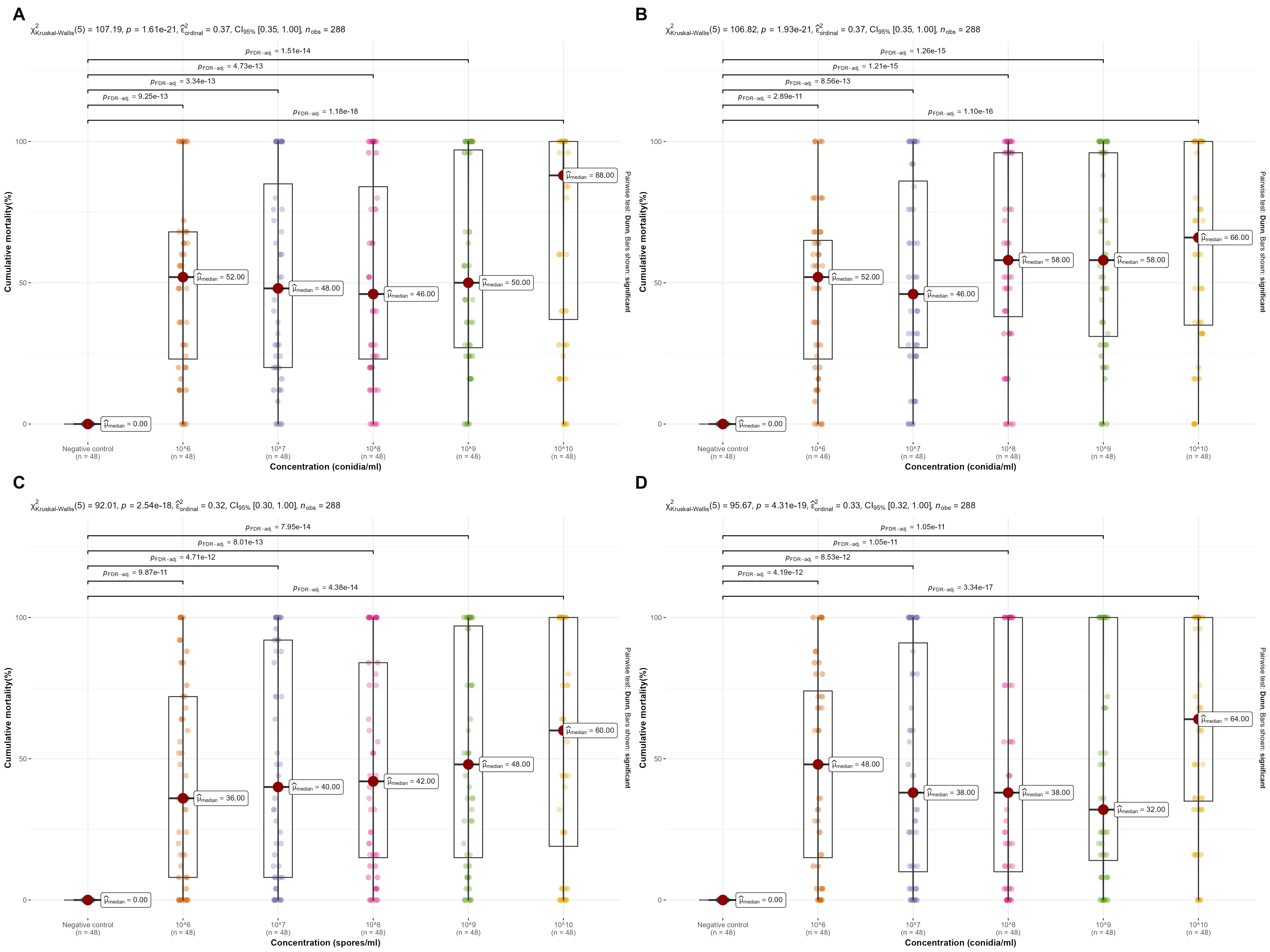

Supplement: Supplementary Figure 8 — Kruskal Wallis multiple comparisons for adult mortality among USJ and NO populations for spores of A. niger and T. atroviride for different concentrations. (A) A. niger on USJ population, (B) A. niger on NO population, (C) T. atroviride on USJ population, (D) T. atroviride on NO population. [file Image8.jpeg]
